# Circadian transcriptome analysis in human fibroblasts from Hunter syndrome and impact of iduronate-2-sulfatase treatment

**DOI:** 10.1186/1755-8794-6-37

**Published:** 2013-10-02

**Authors:** Gianluigi Mazzoccoli, Rosella Tomanin, Tommaso Mazza, Francesca D’Avanzo, Marika Salvalaio, Laura Rigon, Alessandra Zanetti, Valerio Pazienza, Massimo Francavilla, Francesco Giuliani, Manlio Vinciguerra, Maurizio Scarpa

**Affiliations:** 1 Department of Medical Sciences, Division of Internal Medicine and Chronobiology Unit, IRCCS Scientific Institute and Regional General Hospital “Casa Sollievo della Sofferenza”, S.Giovanni Rotondo (FG), Italy; 2 Laboratory of Diagnosis and Therapy of Lysosomal Disorders, Department of Women’s and Children’s Health, University of Padova, Padova, Italy; 3 Bioinformatics Unit, IRCCS Scientific Institute and Regional General Hospital “Casa Sollievo della Sofferenza”, S.Giovanni Rotondo (FG), Italy; 4 Research Laboratory of Gastroenterology Unit, IRCCS Scientific Institute and Regional General Hospital “Casa Sollievo della Sofferenza”, S.Giovanni Rotondo (FG), Italy; 5 Computing Unit, IRCCS Scientific Institute and Regional General Hospital “Casa Sollievo della Sofferenza”, S.Giovanni Rotondo (FG), Italy; 6 Institute for Liver and Digestive Health, Division of Medicine, Royal Free Campus, University College London, London, UK; 7 Centre for Rare Disorders, IRCCS Scientific Institute and Regional General Hospital “Casa Sollievo della Sofferenza”, S.Giovanni Rotondo (FG), Italy

**Keywords:** Clock gene, Hunter syndrome, Lysosomal storage disease, Circadian rhythm

## Abstract

**Background:**

Hunter syndrome (HS) is a lysosomal storage disease caused by iduronate-2-sulfatase (IDS) deficiency and loss of ability to break down and recycle the glycosaminoglycans, heparan and dermatan sulfate, leading to impairment of cellular processes and cell death. Cell activities and functioning of intracellular organelles are controlled by the clock genes (CGs), driving the rhythmic expression of clock controlled genes (CCGs). We aimed to evaluate the expression of CGs and downstream CCGs in HS, before and after enzyme replacement treatment with IDS.

**Methods:**

The expression levels of CGs and CCGs were evaluated by a whole transcriptome analysis through Next Generation Sequencing in normal primary human fibroblasts and fibroblasts of patients affected by HS before and 24 h/144 h after IDS treatment. The time related expression of CGs after synchronization by serum shock was also evaluated by qRT-PCR before and after 24 hours of IDS treatment.

**Results:**

In HS fibroblasts we found altered expression of several CGs and CCGs, with dynamic changes 24 h and 144 h after IDS treatment. A semantic hypergraph-based analysis highlighted five gene clusters significantly associated to important biological processes or pathways, and five genes, *AHR, HIF1A, CRY1, ITGA5* and *EIF2B3,* proven to be central players in these pathways. After synchronization by serum shock and 24 h treatment with IDS the expression of *ARNTL2* at 10 h (p = 0.036), *PER1* at 4 h (p = 0.019), *PER2* at 10 h (p = 0.041) and 16 h (p = 0.043) changed in HS fibroblasts.

**Conclusion:**

CG and CCG expression is altered in HS fibroblasts and IDS treatment determines dynamic modifications, suggesting a direct involvement of the CG machinery in the physiopathology of cellular derangements that characterize HS.

## Background

Hunter syndrome (HS), or Mucopolysaccharidosis type II, is a lysosomal storage disease and a X-linked recessive genetic disorder caused by a mutation in the *IDS* gene, leading to absence or deficiency of the enzyme iduronate-2-sulfatase (IDS). IDS deficit interferes with the ability to break down and recycle glycosaminoglycans (GAGs), precisely dermatan sulfate and heparan sulfate, causes lysosomal engulfment, and hinders the customary working of cellular functions, compromising anatomical integrity in a number of organ systems and leading to curtailment of life expectancy [1]. The patients are characterized by coarsening of facial features, bone and joint abnormalities, short stature, changes in the heart, respiratory system, hearing and vision, and in more severe forms by disturbed motor function, progressive learning difficulties and behavioural abnormalities [2]. Enzyme replacement therapy with idursulfase, a recombinant form of human IDS, represents the only affordable therapeutic approach presently.

The cellular processes in every life form from bacteria to humans show diurnal variations driven by an internal timing system, and the oscillation frequency has a near-24-hour period, so that the rhythmicity is defined circadian (from the latin *circa*, about, and *dies*, a day) [3-5]. The mammalian circadian timing system is composed by a central pacemaker and master oscillator in the suprachiasmatic nuclei (SCN) of the brain and self-sustained oscillators in the peripheral tissues [6,7]. The SCN are entrained to the environmental light/dark cycle by cues perceived through photon capture, signalling by melanopsin-containing retinal ganglion cells, and inputs conveyed by the retino-hypothalamic tract [8]. They synchronize peripheral oscillators and coordinate bodily functions by means of output pathways that may be neural projections (sympathetic and parasympathetic nerve fibers) or diffusible factors (for example hormones as cortisol and melatonin) [9,10].

The molecular mechanisms underlying the biological clock functioning consist of a transcriptional-translational feedback loop operated by a set of genes, called core clock genes, coding for proteins that in turn block gene expression, completing a cycle in approximately 24 hours [11]. The positive limb of the loop is represented by the *CLOCK* (or its paralog *NPAS2*) and *ARNTL/BMAL1* (or its paralog *ARNTL2/BMAL2*) genes that code for the transcription factors CLOCK/NPAS2 and ARNTL/2. A heterodimer of the CLOCK/NPAS2 and ARNTL/2 proteins rhythmically activates the transcription of clock genes *PER1, PER2, PER3, CRY1, CRY2,* coding for PER and CRY proteins that represent the negative limb of the loop. PER and CRY proteins along with Casein Kinase Iδ/ϵ (CKIδ/ϵ) and Casein Kinase II (CKII A1, A2, B), responsible of multilevel posttranslational regulation of various clock components, cooperate to form a repression complex that translocates back into the nucleus, interacts directly with CLOCK/NPAS2 and ARNTL/2, and impede their transcriptional activity [12]. The clock gene machinery is connected to a supporting feedback loop operated by the nuclear receptors REV-ERBα/β (encoded by the genes NR1D1 and NR1D2) and retinoic acid-related orphan receptor (ROR) α/γ, where RORs positively regulate *ARNTL/2* and *REV-ERB* expression and, in turn, REV-ERBs antagonize RORs [13,14]. Besides, SIRT1, a NAD + −dependent protein and histone deacetylase, is required for high-magnitude circadian transcription of several core clock genes, including *ARNTL, PER2*, and *CRY1*. SIRT1 counterbalances the histone acetyltransferase activity of CLOCK, binds CLOCK-ARNTL heterodimer in a circadian manner and promotes the deacetylation and degradation of PER2 [15].

*TIMELESS* is a core circadian clock gene in Drosophila melanogaster and is maintained in mammals, but its role in mammalian circadian clock function is not clear. TIMELESS and its partner TIMELESS interacting protein (TIPIN) interact with components of the DNA replication system to regulate DNA replication processes under both normal and stress conditions and are essential for ataxia telangiectasia and Rad3-related (ATR)-checkpoint kinase (Chk)1 and ataxia telangiectasia mutated (ATM)-checkpoint kinase (Chk)2-mediated signaling and S-phase arrest [16-19].

The clock gene oscillation drives the rhythmic expression of other genes defined clock controlled genes, for example *DBP*, *TEF*, *HLF*, *HSF1*, *NFIL3* (also called *E4BP4*), *DEC1-2* (also called *BHLHE40-41*), which steer downstream tissue specific genes regulating key cellular functions, such as cell cycle progression, proliferation, DNA damage response, autophagy, apoptosis, metabolism, redox equilibrium [20-23], and are involved in physiological processes such as inflammation/immune response [24,25], and paraphysiological phenomena, such as aging [26]. About 5% to 15% of genome-wide mRNA expression exhibits a circadian rhythm that is driven by the clock genes and there is a tissue specificity of cycling genes and a tissue specificity of clock controlled gene expression timing and level (output genes i.e., genes involved in the functional output of an organ) [27-30]. Accordingly, multicellular organisms are characterized by tissue-specific circadian regulation of transcription, with peripheral oscillators controlling the definite biochemical cascades relevant to their tissue or organ function and generating rhythms in various pathways, including those involved in intracellular activities and depending on the correct functioning of intracellular organelles [27-30].

The importance of the role played by the clock gene machinery in the regulation of the processes in the intracellular organelles is confirmed by the physiopathology of Niemann-Pick types A and B disease, related to deficit of sphingomyelin synthase 2 (SGMS2), the enzyme that catalyzes the transfer of phosphocholine from phosphatidylcholine onto ceramide to produce sphingomyelin, a major component of cell and Golgi membranes. Furthermore, Niemann-Pick type C disease is related to deficit of Niemann-Pick C1 (NPC1), which is crucial for the intracellular trafficking of cholesterol from the late endosome to the trans-Golgi network [31]. SGMS2 and NPC1protein levels oscillate with circadian rhythmicity driven by the clock controlled genes *SGMS2* and *NPC1* respectively [27,28]. Patients carrying a mutation in these genes develop a condition characterized by accumulation of sphingomyelin in spleen, liver, lungs, bone marrow and brain, causing irreversible neurological damage, and high cholesterol levels in the endosomal-lysosomal system, respectively [32].

The aim of our study was to assess the expression of clock genes and clock controlled genes in Mucopolysaccharidosis type II, and to evaluate the circadian pattern of variation and the effects of the treatment with idursulfase on the expression of clock genes and clock controlled genes at different time points.

We addressed these issues taking advantage of an *in vitro* model of HS, represented by human fibroblasts with the mutational features of this mucopolysaccharidosis, through evaluation of the mRNA expression levels of the core clock genes and of a panel of clock controlled genes selected by means of literature-mining [27-30,33] (for a complete list of circadian transcripts refer to: CircaDB at http://circadb.org). These evaluations are part of a whole transcriptome analysis performed by Next Generation Sequencing (NGS) in fibroblasts from healthy subjects and fibroblasts from HS patients before, 24 hours and 144 hours after idursulfase treatment. We also evaluated by qRT-PCR the expression levels of core clock genes upon serum-shock induced synchronization in normal human fibroblasts and fibroblasts of patients affected by HS before and after 24 hours of treatment with idursulfase.

## Materials and methods

### Cells

Human fibroblasts from skin biopsy of five HS paediatric patients carrying different mutation in IDS gene were obtained from “Cell Line and DNA Bank from Patients Affected by Genetic Diseases”, Gaslini Institute (Genoa, Italy). As healthy controls human fibroblasts from four children’s circumcision were used; they were obtained from the Histology Unit of the Department of Histology, Microbiology and Medical Biotechnology (University of Padua, Padua, Italy). Written informed consents were obtained from patients at the time of biopsy and the study was approved by the Ethics Committee of the University of Padua, Padua, Italy. All cells were anonymously obtained.

### Cell culture and treatment with idursulfase of HS fibroblasts

Primary fibroblasts were cultured at 37° C in 5% CO2 atmosphere in RPMI medium supplemented with 15% fetal bovine serum (FBS), 100 U/ml penicillin and 100 ng/ml streptomycin (Invitrogen Life Technologies, Milan, Italy). Fibroblasts of patients affected by HS were treated with idursulfase (Elaprase®, Shire Human Genetic Therapies, Inc, Lexington, MA, USA) at a concentration of 62.5 nM for 24 hours and the cells were harvested 24 h and 144 h after idursulfase treatment.

### Whole transcriptome analysis performed by NGS technology

Total RNA was extracted using the TRIzol® Reagent (Sigma-Aldrich®) according to the manufacturer’s protocols. Isolated RNA was quantified by NanoDrop ND-1000 Spectrophotometer (Thermo Scientific, Barnstead, NH, USA) and further assessed for quality using the Agilent 2100 Bioanalyzer (Santa Clara, CA) prior to library construction. Total RNAs extracted from different cell lines were equally pooled into two specimens (Hunter and healthy) using 12.5 μg of RNA from each cell line. From total RNA of each pool mRNA purification was performed trough poliA(+) enrichment by Dynabeads® mRNA Purification Kit (Life Technologies™ Carlsbad, CA, USA). Total RNA was used for standard fragment-library preparation using the SOLiD Total RNA-Seq Kit (Life Technologies). Finally, emulsion PCR reactions were carried out by mixing appropriated amount of libraries with SOLiD beads. After PCR amplification, emulsions were broken using butanol, and the beads were washed, enriched, and terminal transferased before quantification and deposition onto a slide for sequencing. Templated beads were deposited onto one full slide, one sample for quarter (quad). Sequencing was carried out to 35 bases using SOLiD™ 3 (Sequencing by Oligo Ligation and Detection) System and following the manufacturer’s instructions.

### Alignment

Reads obtained from sequencing were aligned on human genome by CRIBI Biotechnology Centre (University of Padova) using the PASS software (http://pass.cribi.unipd.it). The February 2009 human genome assembly (GRCh37), publicly available at UCSC Genome Bioinformatics Site (http://genome.ucsc.edu/) was taken as reference sequence and the options best-hit, gap = 0 and maximun mismach = 3 have been selected. Only unique reads (aligning on only one gene) and only genes with coverage higher than 50% of gene length were considered.

### Serum-shock induced synchronization and treatment with idursulfase of HS fibroblasts

The serum shock induced synchronization was performed as follows: approximately 4×10^5^ cells/6 wells were plated the day before the experiments. Fibroblasts of patients affected by HS were treated with idursulfase at a concentration of 62.5 nM for 24 hours (Elaprase, Shire Human Genetic Therapies, Inc, Lexington, MA, USA). The day of the experiments, culture medium was exchanged with serum-rich medium (RPMI containing 50% FBS) and after 2 hours this medium was replaced with serum free RPMI [34]. The cells were harvested 1 h, 4 h, 10 h, 16 h, 22 h and 28 h after serum shock.

### Quantitative real time polymerase chain reaction (qRT-PCR)

Total RNA was extracted from normal primary human fibroblasts and fibroblasts of patients affected by HS before and after 24 hours of idursulfase treatment at the indicated time points using the RNeasy® Mini Kit (Qiagen S.p.a. Milan, Italy) and subsequently digested by DNase I. Quantitative Real Time PCR was performed starting from 100 ng of purified RNA using the one step quantifast SYBR Green RT PCR KIT (Qiagen). For real-time PCR, we used the following SYBR Green QuantiTect Primers purchased from Qiagen: ARNTL (QT00068250), ARNTL2 (QT00011844), CLOCK (QT00054481), CRY1 (QT00025067) PER1 (QT00069265), PER2 (QT00011207), PER3 (QT00097713). Expression levels of target gene were normalized using the housekeeping control gene TATA binding protein (TBP, QT00000721). Values of mRNA expression levels of clock genes were calculated using the formula 2^−ΔΔCt^.

### Statistical analysis

Results are expressed as means ± SE of at least three different experiments. Comparisons were made using Student’s t-test as appropriate. The limit of statistical significance was set at to p < 0.001 for comparisons of levels of mRNA expression determined by NGS, and p < 0.05 for comparisons of levels of mRNA expression determined by qRT-PCR. Each time series of clock gene expression levels was analyzed for circadian rhythm characteristics by the single cosinor procedure involving the fit of a 24-h cosine curve to the data by least squares linear regression, in order to accurately describe waveforms and rhythm characteristics. An R2 value and a p value for the rejection of the zero-amplitude assumption were determined for each component in the cosine model separately and overall, with rhythm detection considered statistically significant if p ≤ 0.05 and borderline significant if p > 0.05 and p < 0.10 for any period tested. Circadian (24-h) characteristics were summarized from the 24-h cosine. Rhythm characteristics determined from the best-fitting cosine model include the MESOR (the middle of the cosine representing an adjusted average if unequal sampling), the amplitude (half the distance from the peak and trough of the best fitting curve), and the acrophase of the cosine model (the peak of a single component cosine). All analyses were performed using the MATLAB 6.5 statistical package (MathWorks, Natick, MA, USA).

## Results

The mRNA expression levels of the core clock genes *ARNTL, ARNTL2, CLOCK, CRY1, CRY2, CSNK1D, CSNK1E, CSNK2A1, CSNK2A2, CSNK2B, NPAS2, NR1D1*, *NR1D2*, *PER1, PER2, PER3, RORA*, *SIRT1, TIMELESS*, *TIPIN* and of a set of clock controlled genes were evaluated as part of a whole transcriptome analysis performed by NGS technology in healthy human fibroblasts (C) and fibroblasts of HS patients before (H), 24 hours (T1) and 144 hours (T2) after idursulfase treatment. Ratio, log2 (ratio), fold change and p-values are shown in Additional file 1: Table S1.

The expression levels of *ARNTL, ARNTL2, CLOCK, CRY1, PER1, PER2,* and *PER3* were also evaluated by qRT-PCR after serum-shock induced synchronization in normal human fibroblasts and fibroblasts of patients affected by HS before and after 24 hours of treatment with idursulfase.

### Evaluation by NGS technology of core clock gene expression in HS fibroblasts vs healthy fibroblasts (H vs C)

In HS fibroblasts the genes *CLOCK* (fold change = −1.45, p < 0.0001), *NPAS2* (fold change = −1.65, p < 0.0001), *CRY1* (fold change = −1.51, p < 0.0001), *NR1D2* (fold change = −1.97, p < 0.0001) and *SIRT1* (fold change = −1.30, p < 0.0001) showed lower expression levels compared to healthy controls. Conversely the genes *PER1* (fold change = 1.27, p < 0.0001), *CSNK1D* (fold change = 1.26, p < 0.0001), *CSNK1E* (fold change = 1.54, p < 0.0001), and *NR1D1* (fold change = 1.99, p < 0.0001) showed higher expression levels in HS fibroblasts. The expression level of *ARNTL* (fold change = 1.34, p = 0.01), *ARNTL2* (fold change = 1.09, p = 0.47), *PER2* (fold change = 1.40, p = 0.001), *PER3* (fold change = −1.01, p = 0.79), *CRY2* (fold change = 1.16, p = 0.02), *CSNK2A1* (fold change = 1.02, p = 0.56), *CSNK2A2* (fold change = −1.08, p = 0.26), *RORA* (fold change = −1.07, p = 0.28), *TIMELESS* (fold change = 1.25, p = 0.006), and *TIPIN* (fold change = −1.25; p = 0.27) was not different in a statistically significant way between normal and HS fibroblasts or did not reach the p < 0.001 threshold value.

### Evaluation by NGS technology of core clock gene expression levels in HS fibroblasts after 24 hours of treatment with idursulfase versus healthy fibroblasts (T1 vs C)

In HS fibroblasts after 24 hours of treatment with idursulfase *NPAS2* (fold change = −1.39, p = 0.0005), *CRY1* (fold change = −1.75, p < 0.0001) and *NR1D2* (fold change = −1.65, p < 0.0001) genes showed lower expression levels in comparison to controls. The genes *ARNTL* (fold change = 1.53, p < 0.0001), *ARNTL2* (fold change = 2.22, p < 0.0001), *PER2* (fold change = 1.72, p < 0.0001), *CSNK1E* (fold change = 1.47, p < 0.0001), *CSNK2B* (fold change = 1.15, p < 0.0001), *NR1D1* (fold change = 3.59, p < 0.0001), and *TIMELESS* (fold change = 2.41, p < 0.0001) showed higher expression levels in HS fibroblasts after 24 h treatment, in comparison to healthy human fibroblasts.

The expression level of *CLOCK* (fold change = −1.13, p = 0.003), *PER1* (fold change = 1.22, p = 0.004), *PER3* (fold change = 1.14, p = 0.03), *CRY2* (fold change = 1.04, p = 0.55), *CSNK2A1* (fold change = 1.04, p = 0.30), *CSNK1D* (fold change = 1.12, p = 0.007), *CSNK2A2* (fold change = −1.09, p = 0.25), *RORA* (fold change = −1.14, p = 0.03), *SIRT1* (fold change = −1.12, p = 0.07) and *TIPIN* (fold change = 1.4; p = 0.03) was not different in a statistically significant way between normal and idursulfase treated HS fibroblasts or did not reach the p < 0.001 threshold value.

### Evaluation by NGS technology of core clock gene expression levels in HS fibroblasts 144 hours after treatment with idursulfase versus healthy fibroblasts (T2 vs C)

In HS fibroblasts 144 hours after treatment with idursulfase the genes *CLOCK* (fold change = −1.66, p < 0.0001), *NPAS2* (fold change = −1.56, p < 0.0001), *CRY1* (fold change = −2.00, p < 0.0001), *NR1D2* (fold change = −2.53, p < 0.0001), *RORA* (fold change = −1.28, p = 0.0003), *TIMELESS* (fold change = −1.93, p < 0.0001) and *SIRT1* (fold change = −1.41, p < 0.0001) showed lower expression levels in comparison to controls. *CSNK1E* (fold change = 1.27, p < 0.0001), and *NR1D1* (fold change = 1.72, p < 0.0001) showed higher expression levels in HS fibroblasts 144 hours after idursulfase treatment in comparison to healthy human fibroblasts. The expression level of *ARNTL* (fold change = 1.38, p = 0.007), *ARNTL2* (fold change = −1.36, p = 0.03), *PER1* (fold change = 1.02, p = 0.70), *PER2* ((fold change = 1.20 p = 0.10), *PER3* (fold change = −1.08, p = 0.25), *CRY2* (fold change = 1.05, p = 0.48), *CSNK1D* (fold change = 1.11, p = 0.01), *CSNK2A1* (fold change = −1.14, p = 0.02), *CSNK2A2* (fold change = −1.29, p = 0.002), *CSNK2B* (fold change = 1.00, p = 0.85), and *TIPIN* (fold change = −1.64; p = 0.03) was not different in a statistically significant way between normal and idursulfase treated HS fibroblasts or did not reach the p < 0.001 threshold value.

### Evaluation by NGS technology of changes of clock gene expression levels in HS fibroblasts after 24 hours of treatment with idursulfase versus untreated HS fibroblasts (T1 vs H)

In HS fibroblasts after 24 hours of treatment with IDS *CSNK2B* (fold change = −1.60, p < 0.0001) showed lower expression levels compared to untreated HS fibroblasts (H). Conversely the genes *CLOCK* (fold change = 1.28,p < 0.0001), *ARNTL2* (fold change = 2.03, p < 0.0001), *NR1D1* (fold change = 1.80, p < 0.0001), *NR1D2* (fold change = 1.19, p < 0.001), and *TIMELESS* (fold change = 1.91, p < 0.0001) showed higher expression levels in HS fibroblasts treated for 24 hours with idursulfase , in comparison to untreated HS fibroblasts. The expression level of *NPAS2* (fold change = 1.18, p = 0.11), *ARNTL1* (fold change = 1.14, p = 0.21), *PER1* (fold change = −1.04, p = 0.50), *PER2* (fold change = 1.23, p = 0.02), *PER3* (fold change = 1.16, p = 0.02), *CRY1* (fold change = −1.16, p = 0.09), *CRY2* (fold change = −1.11, p = 0.11), *CSNK1D* (fold change = −1.12, p = 0.05), *CSNK1E* (fold change = −1.05, p = 0.20), *CSNK2A1* (fold change = 1.01, p = 0.66), *CSNK2A2* (fold change = −1.00, p = 0.98), *RORA* (fold change = −1.07, p = 0.30), *SIRT1* (fold change = 1.16, p = 0.04), and *TIPIN* (fold change = 1.83; p = 0.01) was not different in a statistically significant way between treated and not treated HS fibroblasts or did not reach the p < 0.001 threshold value.

### Evaluation by NGS technology of changes of clock gene expression levels in HS fibroblasts after 144 hours of treatment with idursulfase versus untreated HS fibroblasts (T2 vs H)

In HS fibroblasts after 144 hours of treatment with IDS the genes *CSNK1E* (fold change = −1.12, p < 0.0001), *CSNK2A1* (fold change = −1.17, p < 0.001), *CSNK2B* (fold change = −1.21, p < 0.0001), *NR1D2* (fold change = −1.28, p < 0.0001), and *TIMELESS* (fold change = −2.4, p < 0.0001) showed lower expression levels, in comparison to untreated HS fibroblasts.

The expression level of *CLOCK* (fold change = −1.14, p = 0.009), *NPAS2* (fold change = 1.05, p = 0.64), *ARNTL* (fold change = 1.03, p = 0.78), *ARNTL2* (fold change = −1.48, p = 0.004), *PER1* (fold change = −1.24, p = 0.002), *PER2* (fold change = −1.16, p = 0.14), *PER3* (fold change = −1.06, p = 0.37), *CRY1* (fold change = −1.32, p = 0.002), *CRY2* (fold change = −1.10, p = 0.15), *CSNK1D* (fold change = −1.13, p = 0.004), *CSNK2A2* (fold change = −1.18, p = 0.04), *NR1D1* (fold change = −1.16, p = 0.01), *RORA* (fold change = −1.19, p = 0.01), *SIRT1* (fold change = −1.08, p = 0.33), and *TIPIN* (fold change = −1.31; p = 0.26) was not different in a statistically significant way between treated and not treated HS fibroblasts or did not reach the p < 0.001 threshold value.

### Evaluation by NGS technology of clock controlled gene expression in healthy fibroblasts, untreated HS fibroblasts and HS fibroblasts 24 hours and 144 hours after treatment with idursulfase

Regarding to the clock controlled genes, in HS fibroblasts there was a statistically significant difference and dynamic change with idursulfase treatment at the time points considered of the expression of a huge number of output genes involved in the control of important cellular and tissue processes. The cellular processes driven by clock controlled genes differentially expressed with a statistical significance reaching the p < 0.001 threshold are represented by DNA transcription (*AHR, ARNT, CTNNB1, FOS, FOXL2, FOXO1, HIF1A, HSF1, ID2, JUN, KEAP1, KITLG, KLF10, NFIL3, PPARA, PPARD, PPARG, RARA, RXRA, SMAD4, SP1, SREBF1, SRF, STAT1, STAT5A*), post-translational modification and degradation (*FBXL3, SUMO3, USP5*), biosynthesis (*ACSL1, AEBP1, ALAS1, APBB1, APBB1IP, APBB2, BMPR1A, CEBPB, CREBBP, FASN, GYS1, HMGCR, INSIG2, LPIN1, NAMPT*), processing (*ADA, ALDH1A1, ALDH1A3, ALDH1B1, ATF2, ATM, ATR, ATRIP, BHLHE40, BHLHE41, BNIP3, CES1, CES2, CYP7B1, E2F1, EGFR, EGR1, EIF2B3, HSP90AA1, HSPA1A, HSPA5, HSPD1, GABARAPL1, GLO1, GLUL, HK1, LDHA, LMAN1, LMAN2, MAOA, NPC1, PARP1, PCK2, PRDX2, PYGL, SGMS2, ULK1, XBP1*), transport (*AP2A1, AP2M1, IGFBP3, IGFBP5, LDLR, SLC22A15, SLC25A1, SLC27A1, SLC7A8, SLC9A3R2, SLC9A9*), DNA damage response (*ATM, ATR, ATRIP),* and cell cycle control (*CCNA2, CCNB1, CCND1, CDK2AP1, CDKN1A, GADD45A, MDM2,WEE1*). The tissue processes influenced by deregulated clock controlled genes are mainly represented by inflammation, hemocoagulation and fibrinolysis (*ADAM17, A2M, FN1, ICAM1, IL6, ITGA5, MASP1, MGST1, NFKBIA, PDGFRA, PDGFRB, PTGS2, SERPINE1, SPP1, THBD, TFPI2, TRAF2, VEGFA*).

### Evaluation by qRT-PCR after Serum-Shock Induced Synchronization of changes of clock gene expression levels in healthy fibroblasts, untreated HS fibroblasts and HS fibroblasts after 24 hours of treatment with idursulfase

**Figure 1:**
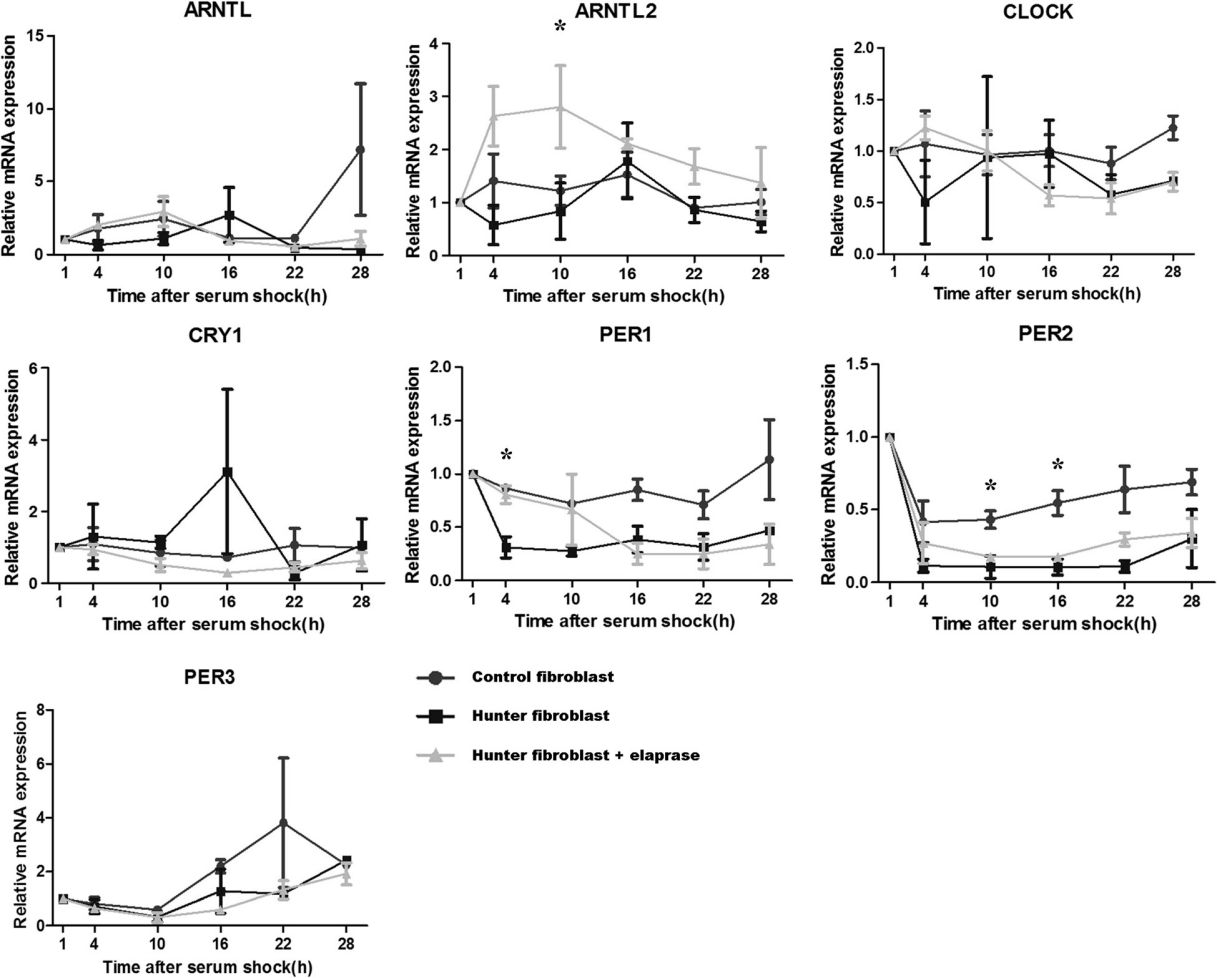
***x-y *plots representing the time related profiles of expression of *ARNTL, ARNTL2, CLOCK, CRY1 PER1, PER2*, and *PER3 *after synchronization with serum shock in control fibroblasts and fibroblasts of patients affected by Hunter Syndrome before and after treatment with iduronate-2-sulfatase.** Original units standardized to T0 (1 h after serum shock) and combined for analyses. * p < 0.05 for ANOVA followed by all pairwise multiple comparison procedures with Student-Newman-Keuls Method.

**Figure 2:**
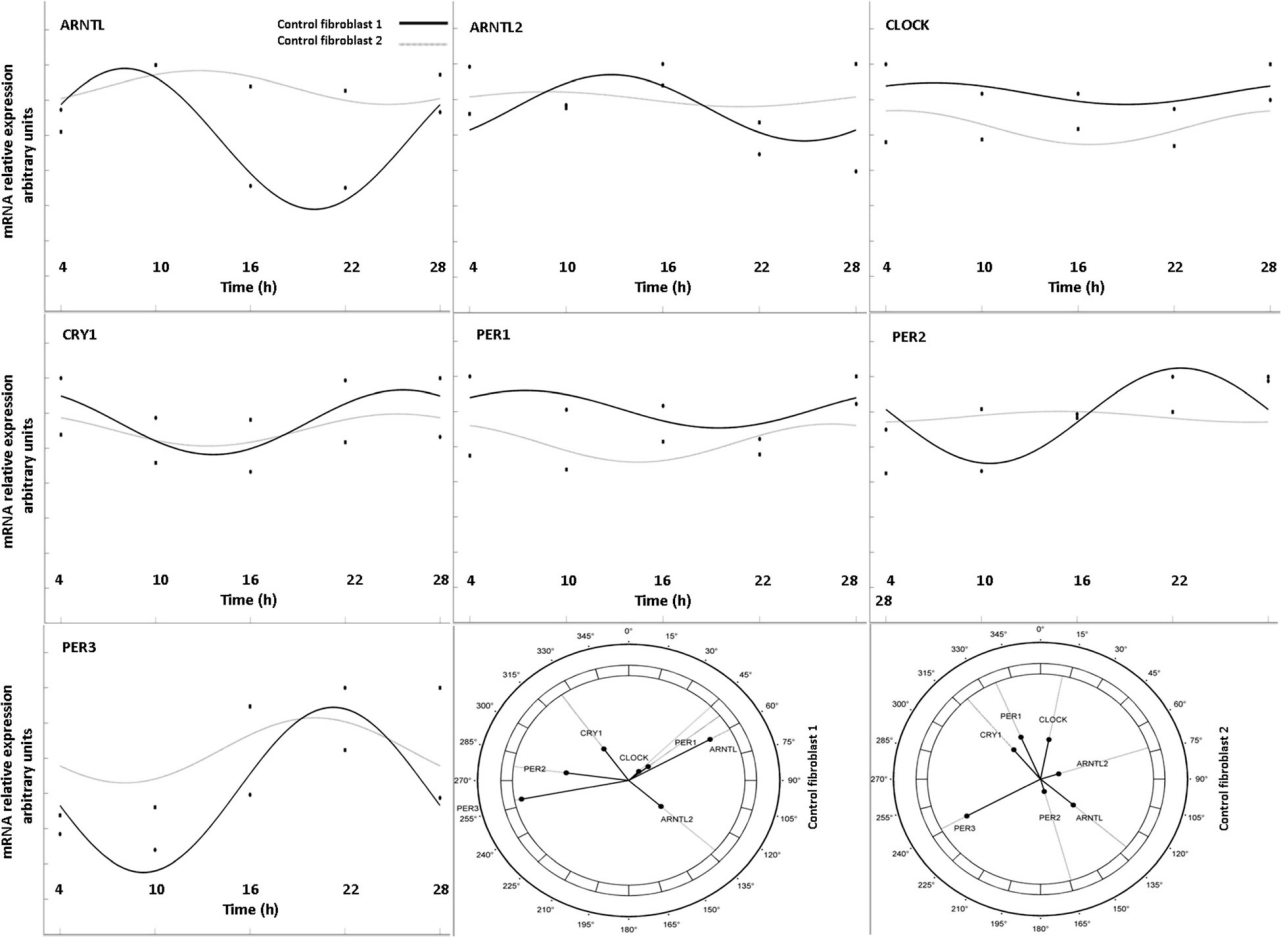
***x-y *plots showing from top to bottom the fitted cosine curves (continous line) superimposed on raw data (squares and circles) and polarograms of *ARNTL, ARNTL2, CLOCK, CRY1 PER1, PER2*, and *PER3 *expression levels after synchronization with serum shock in control fibroblasts.** Original units standardized to T0 (1 h after serum shock) and combined for analyses. Polarograms of cosinor analysis show the acrophases for the clock gene expression values. Radial axis represents the time point (in degrees) after serum shock corresponding to the acme and vector length represents the amplitude of the oscillation.

**Figure 3:**
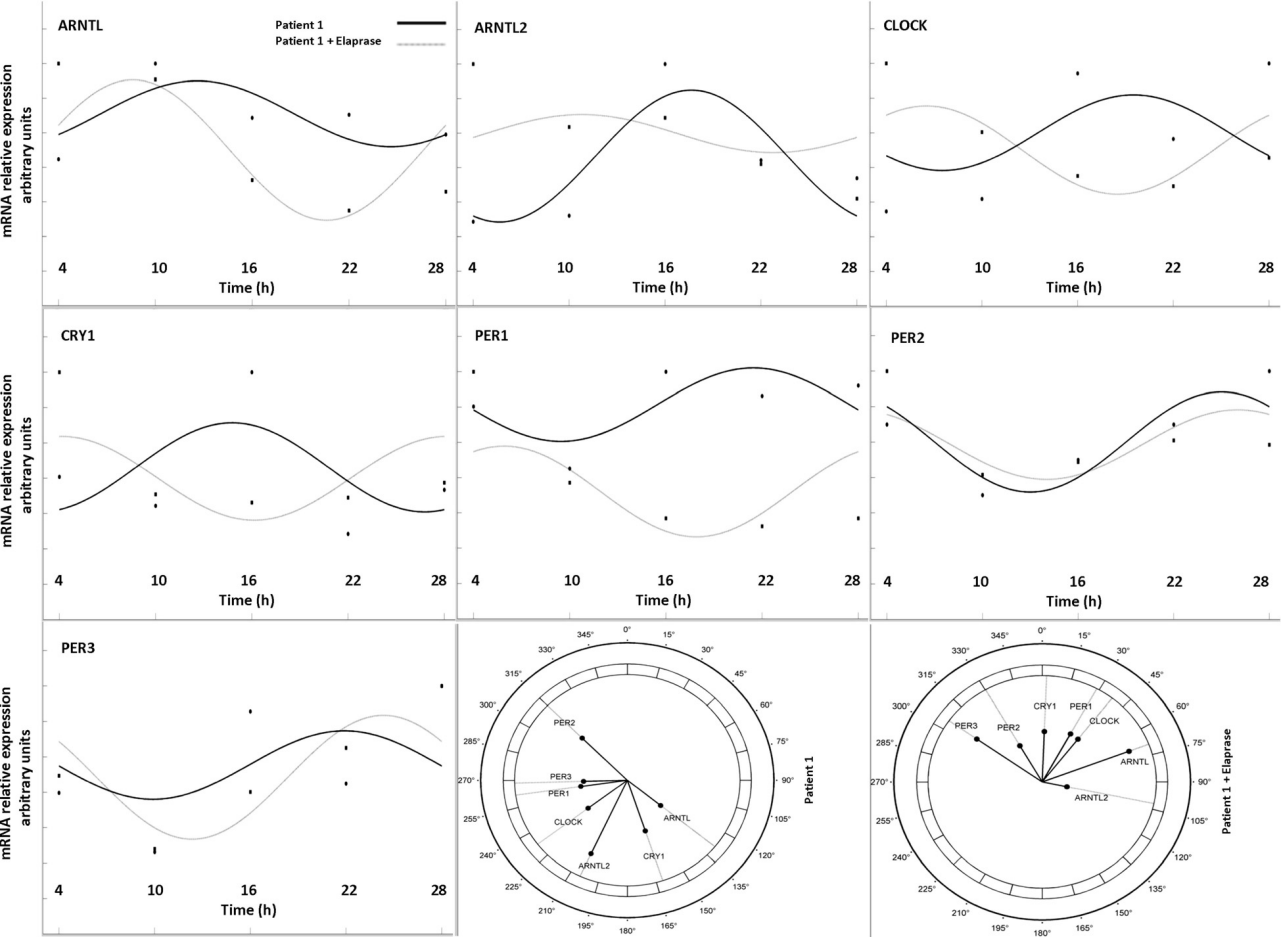
***x-y *plots showing from top to bottom the fitted cosine curves (continous line) superimposed on raw data (squares and circles) and polarograms of *ARNTL, ARNTL2, CLOCK, CRY1 PER1, PER2*, and *PER3 *expression levels after synchronization with serum shock in the fibroblasts of a patient affected by Hunter Syndrome before and after 24-h treatment with iduronate-2-sulfatase.** Original units standardized to T0 (1 h after serum shock) and combined for analyses. Polarograms of cosinor analysis show the acrophases for the clock gene expression values. Radial axis represents the time point (in degrees) after serum shock corresponding to the acme and vector length represents the amplitude of the oscillation.

**Figure 4:**
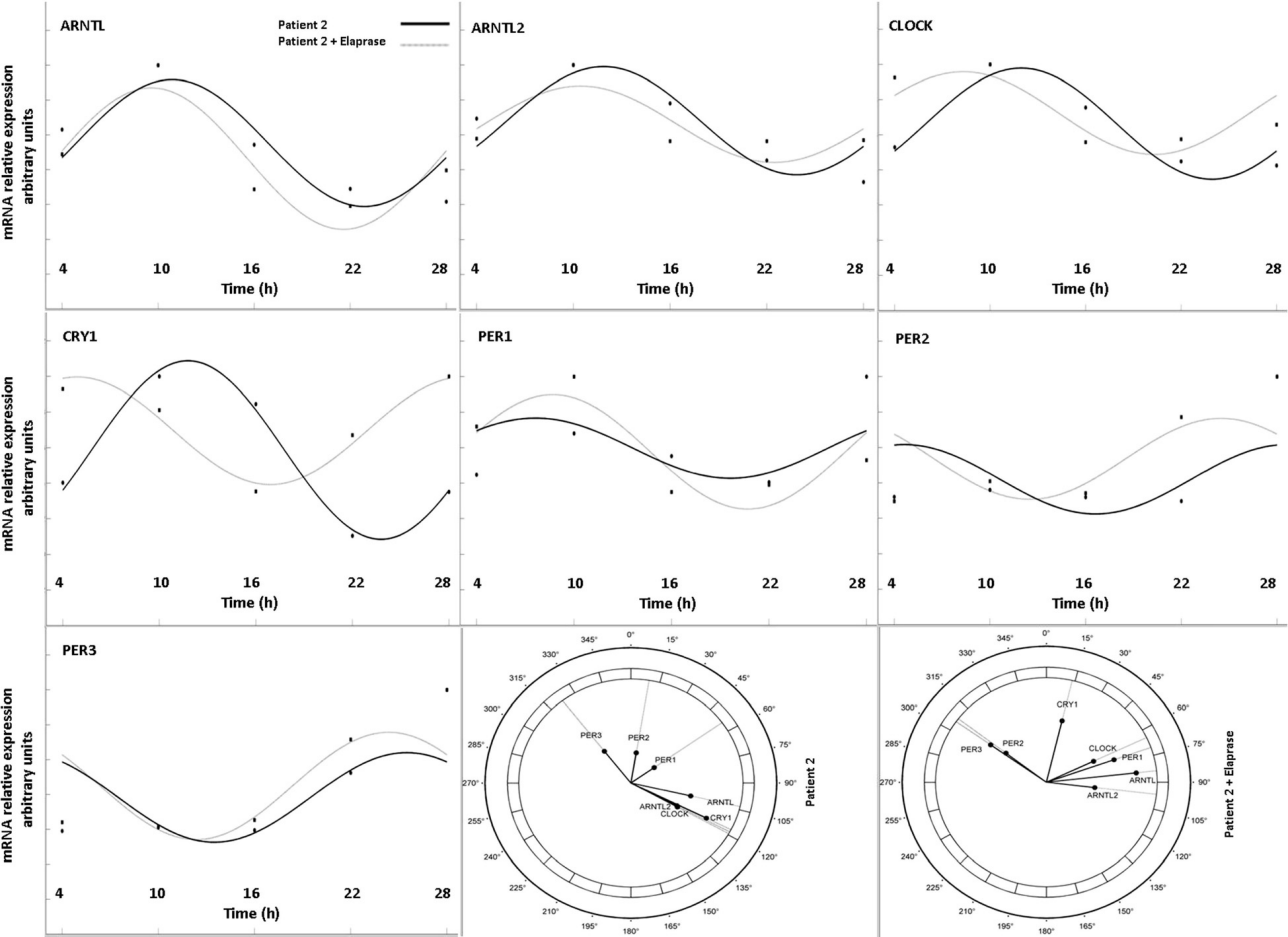
***x-y *plots showing from top to bottom the fitted cosine curves (continous line) superimposed on raw data (squares and circles) and polarograms of *ARNTL, ARNTL2, CLOCK, CRY1 PER1, PER2, *and *PER3 *expression levels after synchronization with serum shock in the fibroblasts of a patient affected by Hunter Syndrome before and after 24-h treatment with iduronate-2-sulfatase.** Original units standardized to T0 (1 h after serum shock) and combined for analyses. Polarograms of cosinor analysis show the acrophases for the clock gene expression values. Radial axis represents the time point (in degrees) after serum shock corresponding to the acme and vector length represents the amplitude of the oscillation.

After serum shock synchronization and qRT-PCR analysis, statistically significant differences were evidenced in the mRNA expression levels of *ARNTL2* at 10 h (p = 0.036), *PER1* at 4 h (p = 0.019), *PER2* at 10 h (p = 0.041) and 16 h (p = 0.043), between idursulfase treated HS fibroblasts and untreated HS fibroblasts (Figure 1). Fitting of cosine curves with a 24-hour period to raw data and plotting as polarograms clock gene expression levels in control fibroblasts and fibroblasts of patients affected by HS before and after 24 hours of idursulfase treatment (Figures 2, 3 and 4) evidenced statistically significant and borderline significant rhythms of clock gene expression, and advance of phase oscillation after idursulfase treatment. MESOR, amplitude, acrophase and p values are reported in Table 1.

**Table 1:** Parameters calculated by Cosinor analysis (fitting a cosine curve with a 24-hour period to raw data) of clock gene expression levels after serum-shock induced synchronization in normal fibroblasts and fibroblasts of patients affected by Hunter syndrome before and after 24 hours of treatment with idursulfase

**Fibroblast of control subject 1**					**Fibroblast of control subject 2**				
	**MESOR**	**Amplitude**	**Acrophase**	**P**		**MESOR**	**Amplitude**	**Acrophase**	**P**
**ARNTL**	0.578	0.401	60° 42′	0.048	**ARNTL**	0.872	0.095	130° 30′	0.747
**ARNTL2**	0.753	0.186	131° 58′	0.734	**ARNTL2**	0.802	0.041	72° 33′	0.948
**CLOCK**	0.834	0.061	45° 00′	0.749	**CLOCK**	0.642	0.096	11° 11′	0.827
**PER1**	0.813	0.106	51° 40′	0.592	**PER1**	0.621	0.108	336° 40′	0.818
**PER2**	0.777	0.271	277° 45′	0.234	**PER2**	0.772	0.030	164° 21′	0.983
**PER3**	0.420	0.469	258° 57′	0.136	**PER3**	0.645	0.185	241° 35′	0.825
**CRY1**	0.747	0.184	324° 45′	0.577	**CRY1**	0.704	0.091	319° 46′	0.842
**Fibroblast of Hunter syndrome patient 1 not treated**					**Fibroblast of Hunter syndrome patient 1 treated with idursulfase**				
	**MESOR**	**Amplitude**	**Acrophase**	**P**		**MESOR**	**Amplitude**	**Acrophase**	**P**
**ARNTL**	0.709	0.190	139° 04′	0.493	**ARNTL**	0.499	0.406	69° 11′	0.444
**ARNTL2**	0.466	0.383	204° 48′	0.280	**ARNTL2**	0.597	0.110	101° 49′	0.928
**CLOCK**	0.600	0.218	232° 42′	0.825	**CLOCK**	0.500	0.255	37° 52′	0.492
**PER1**	0.813	0.207	261° 52′	0.516	**PER1**	0.321	0.257	28° 38′	0.677
**PER2**	0.600	0.282	315° 00′	0.294	**PER2**	0.585	0.196	330° 15′	0.509
**PER3**	0.553	0.193	268° 32′	0.867	**PER3**	0.484	0.350	305° 17′	0.390
**CRY1**	0.461	0.253	161° 40′	0.643	**CRY1**	0.399	0.237	2° 15′	0.593
**Fibroblast of Hunter syndrome patient 2 not treated**					**Fibroblast of Hunter syndrome patient 2 treated with idursulfase**				
	**MESOR**	**Amplitude**	**Acrophase**	**P**		**MESOR**	**Amplitude**	**Acrophase**	**P**
**ARNTL**	0.552	0.365	103° 12′	0.298	**ARNTL**	0.462	0.407	83° 41′	0.163
**ARNTL2**	0.680	0.310	114° 09′	0.260	**ARNTL2**	0.660	0.219	96° 41′	0.349
**CLOCK**	0.662	0.316	119° 17′	0.078	**CLOCK**	0.723	0.235	64° 40′	0.296
**PER1**	0.597	0.169	54° 28′	0.713	**PER1**	0.576	0.323	70° 31′	0.235
**PER2**	0.420	0.196	9° 22′	0.708	**PER2**	0.535	0.229	307° 31′	0.682
**PER3**	0.383	0.257	322° 27′	0.664	**PER3**	0.449	0.308	305° 32′	0.572
**CRY1**	0.585	0.502	116° 45′	0.009	**CRY1**	0.694	0.302	13° 30′	0.039

Cosinor, least-squares fit of single component cosine to all data. MESOR, rhythm-adjusted overall 24 h mean; Amplitude, peak-trough range of cosine model ; Acrophase, peak of cosine in degrees.

### Semantic hypergraph-based analysis of circadian gene expression

The aim of this analysis was to determine the functional groups of genes that play any key-role in the development of HS. The analysis workflow is here preliminary sketched: (i) *RNA-Sequencing*: Detection of core clock gene and clock controlled gene expression profiles; (ii) *Descriptive analysis and feature selection*: Selection of the most varying genes by the ensemble of principal component analysis and linear discriminant analysis; (iii) *Qualitative hypergraph building*: Inference of weighted and signed hypergraphs; (iv) *Quantitative undirected graph construction*: Mathematical agglomeration of weights over the edges; (v) *Weighted topology indices calculation*: Computation of several weighted topology indices for each node and normalization of individual fold changes by the most discriminant index; (vi) *Determination of the strongest connected components*: Hierarchical clustering and matching with critical biological functions [35,36].

### Descriptive analysis and features selection

**Figure 5:**
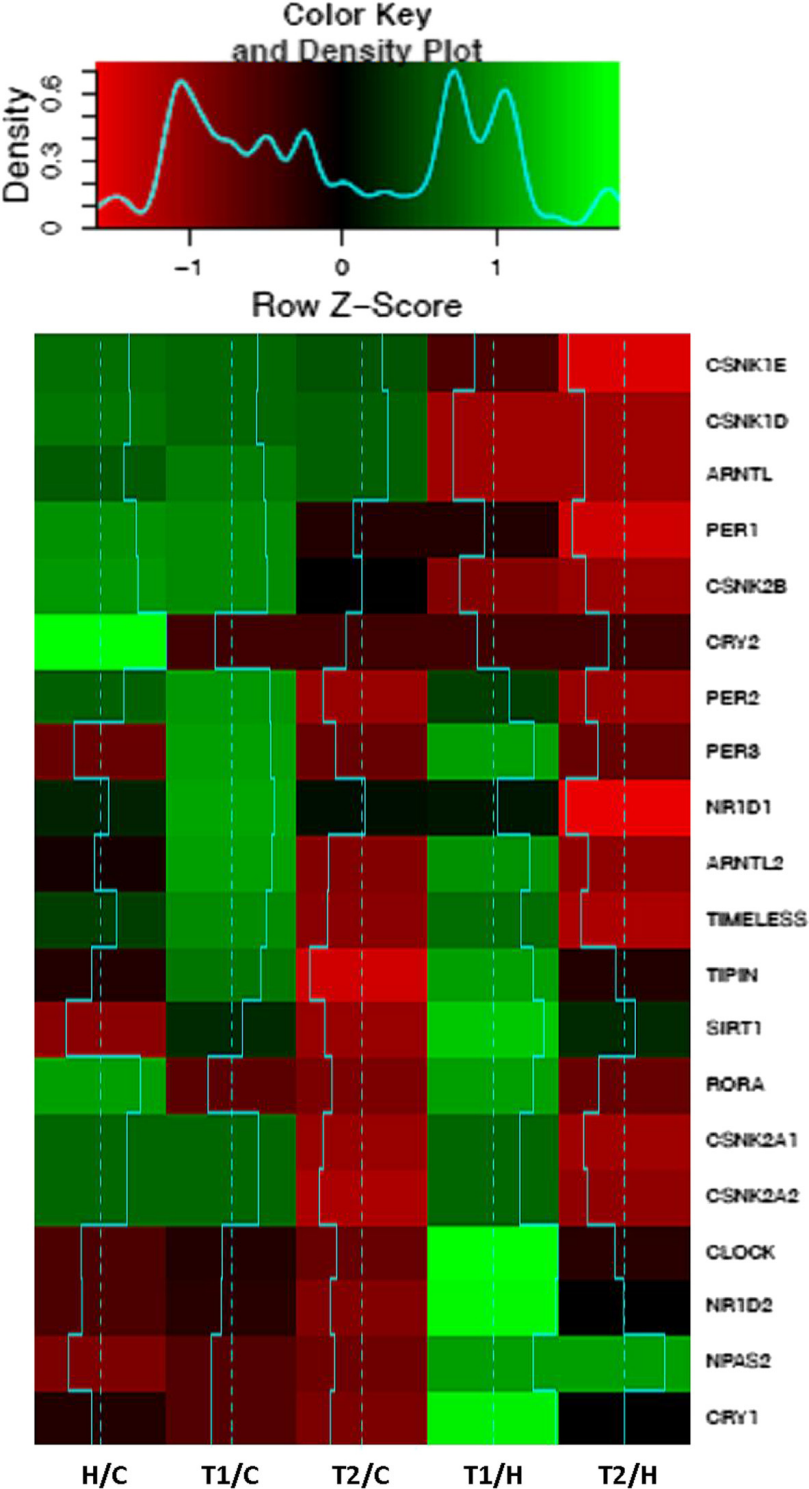
**Heat map of the core clock gene expression fold changes between untreated patients and control cases (H/C), patients 24 or 144 hours after treatment and control cases (T1/C and T2/C), treated and untreated patients (T1/H and T2/H).** Cyan segments and a density plot quantify the magnitude of intra class and global FCs, respectively.

**Figure 6:**
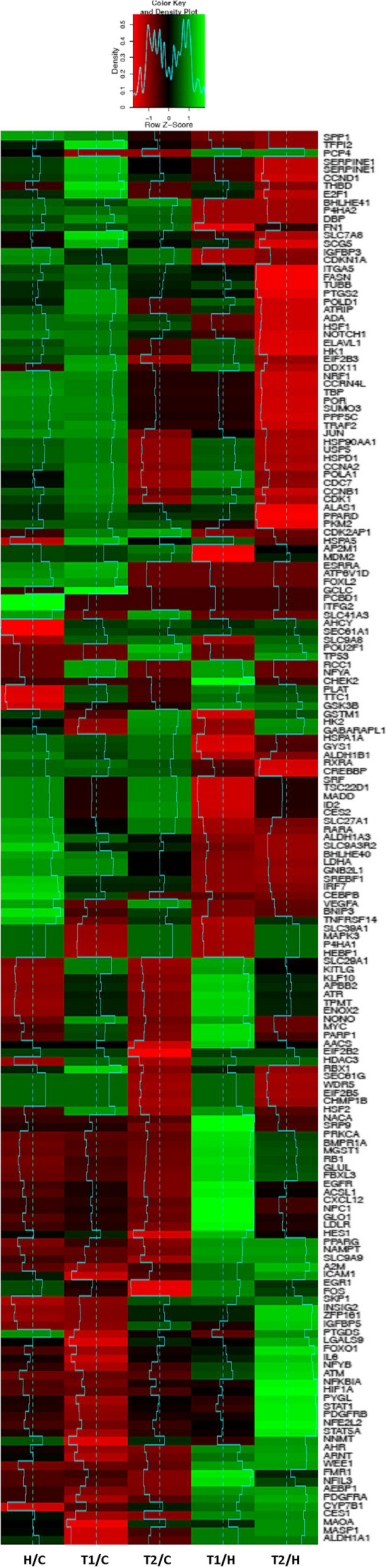
**Heat map of the clock-controlled gene expression fold changes between untreated patients and control cases (H/C), patients 24 or 144 hours after treatment and control cases (T1/C and T2/C), treated and untreated patients (T1/H and T2/H).** Cyan segments and a density plot quantify the magnitude of intra class and global FCs, respectively.

We have preliminarily assessed the expression variation of clock genes and clock-controlled genes, through five different states: HS (H) vs. Control (C), Treatment at time 1 (T1) vs. C, Treatment at time 2 (T2) vs. C, and T1 vs. H, T2 vs. H. We discarded genes that did not exhibit any relevant expression change between these states. We combine a principal component analysis to determine those genes that maximally vary through the first three components, and a linear discriminant analysis to project the genes and their expressions onto the most varying components’ axes (Figures 5 and 6).

### Qualitative hypergraphs building

We have assembled a hypergraph connecting genes by querying and merging a number of heterogeneous data sources: (i) Interpro and PFAM (protein domains), (ii) Gene Expression Omnibus (Co-localization and co-expression), (iii) BIOGRID and IREF (genetic interactions), (iv) PathwayCommons, IMID, NCI_NATURE, REACTOME, KEGG and BIOCARTA (pathways), (v) BIOGRID, BIND, HPRD, INTACT, MINT, MPPI and OPHID (physical interactions) and (VI) curated literature (predicted interactions).Two genes were connected by an edge, whenever at least an interaction evidence of any of the above mentioned interaction categories was found. Several pair of genes resulted to be connected by more than one edge. We built a weighted and signed hypergraph with weights over the edges (carrying the reliability of the corresponding interactions) and weights over the nodes (carrying the fold change expression of the corresponding genes) [37-39].

### Quantitative undirected graph

We deterministically inferred an undirectedm graph from our hypergraph, by applying an injective function to the sets of weights over the edges connecting any two pairs of genes. This simple agglomerative weighting function takes the weights of the edges connecting any two nodes in input, and gives a unique value in output. Constitutively, it gives more and more importance to the pairs that are connected by multiple edges.

$$W_{\mathrm{AB}}=\frac{e^{n}}{n}\sum_{i=1}^{n}W_{AB_{i}}$$

where *n* is the number of edges connecting any two nodes *A* and *B*, and *i* refers to the *ith* edge.

**Figure 7:**
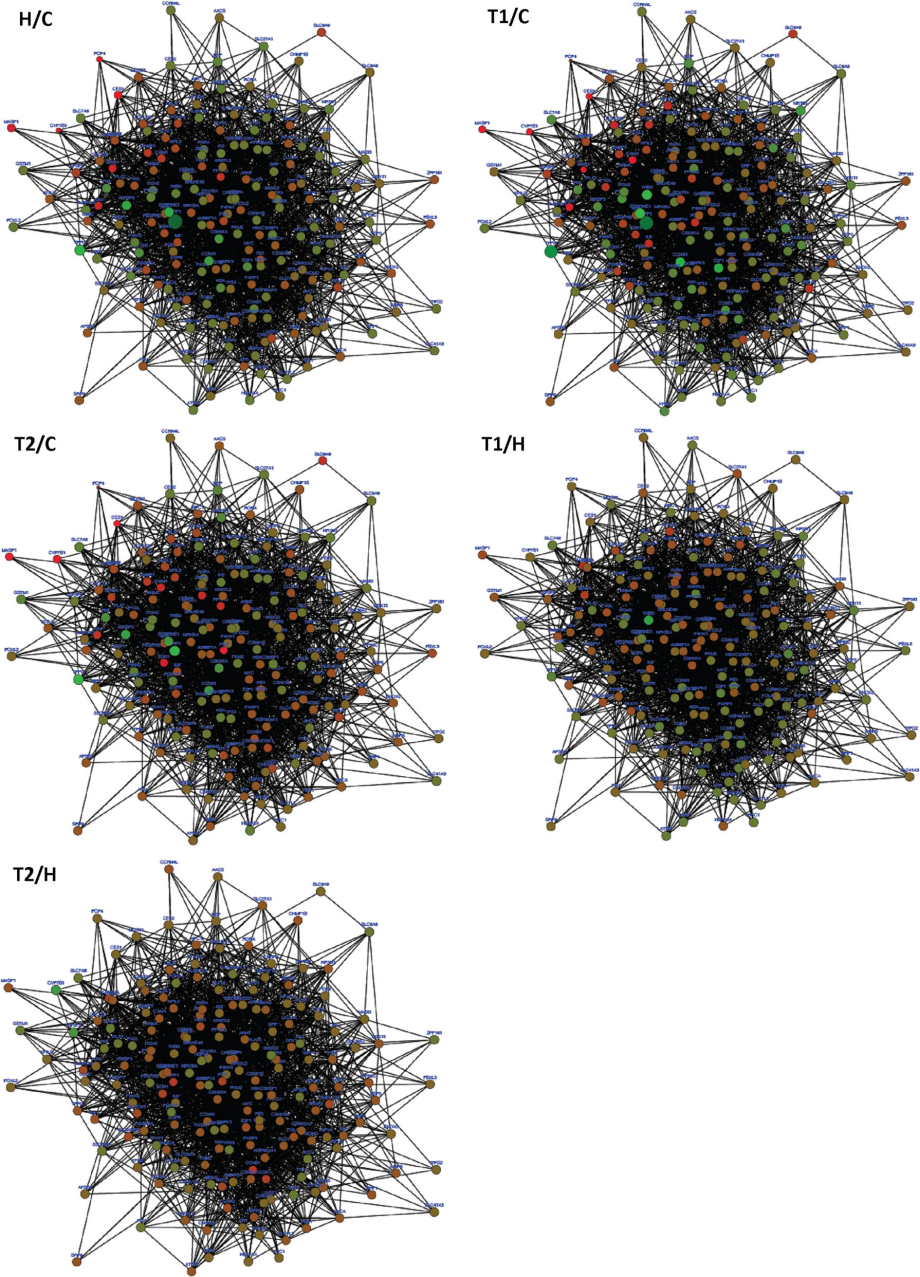
**Undirected and weighted interaction network between core clock genes and clock controlled genes in five different states: Hunter disease (H) vs. Control (C) (H/C), Treatment at time 1 (T1) vs. C (T1/C), Treatment at time 2 (T2) vs. C (T2/C) and T1 vs. H (T1/H), T2 vs. H (T2/H).** Nodes colors range from red (negative FC) to green (positive FC). Diameter of nodes is proportional to the magnitude of the FC. Thickness of edges varies with the reliability of the interaction. Overall, the network exhibits the following global topological properties: clustering coefficient = 0.293, connected components = 1, diameter = 4, radius = 2, centralization = 0.259, shortest paths = 35910 (100%), characteristic path length = 2.002, average number of neighbors = 25.484, density = 0.135 and heterogeneity = 0.498.

Thus, we obtain a new graph that contains the same set of nodes, and individual edges connecting any two nodes, with the newly calculated weights over them (Figure 7).

### Weighted topology indices calculation

We calculated the *closeness* topology index for each gene. We recall that the un-weighted formulation of *closeness* between any two nodes of a graph measures the length of their *shortest paths*. The concept of closeness originates from the inverse definition of *farness,* namely as the inverse of the sum of the distance from a node to all other nodes. Thus, the more important a node is, the lower is its total distance to all other nodes. Closeness can be regarded as a measure of how fast it will take to spread information from a node to all other nodes sequentially. Contrarily, the weighted formulation of closeness takes into account paths whose lengths are not the sum of the minimum number of hops from a node to all other nodes, but the sum of the inverse weights over the edges of the shortest paths from a node to all other nodes. This formulation gives importance to the reliability of a connection, when determining the essentiality of each node, thereby discriminating between reliable and not-reliable interaction paths.

**Figure 8:**
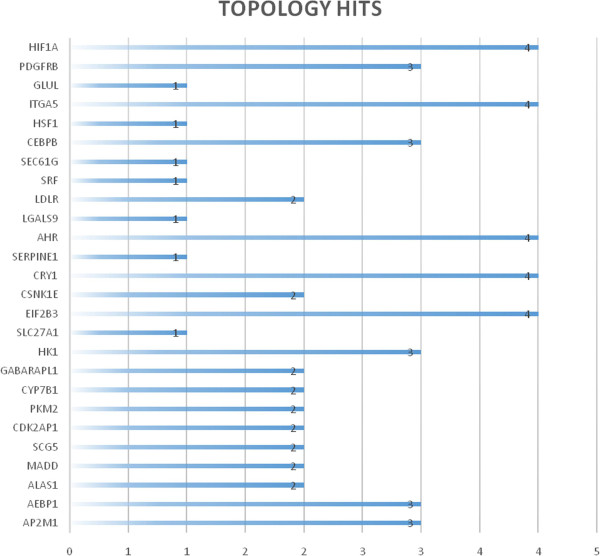
**Genes with the highest topological scores. ***AHR, HIF1A, CRY1, EIF2B3*, and *ITGA5* are among the top 26 most ranked genes by 4 out of 5 indices.

We have subjected the top 26 *closest* genes to other well-known topological indices (i.e., *degree, betweenness, clustering coefficient and topological importance*) and found that 5 (i.e., *AHR, HIF1A, CRY1, ITGA5* and *EIF2B3*, see the plot below) were highly ranked by 4 out of 5 indices (Table 2 and Figure 8).

**Table 2:** The top 26 closest genes subjected to other well-known topological indices (i.e., degree, betweenness, clustering coefficient and topological importance)

**Gene**	**Degree**	**Gene**	**Betweenness**	**Gene**	**Closeness**	**Gene**	**Clustcoefficient**	**Gene**	**Top Importance**
**CDK1**	1.16518997973136	**AEBP1**	0.0391432466904165	**AP2M1**	24.1307228971342	**CCNB1**	0.667477524717011	HIF1A	2.82727459653061
**ARNTL**	0.62961382177992	**GABARAPL1**	0.0337159695650262	**AEBP1**	24.0044419343927	**WEE1**	0.327773492337581	TP53	2.2936294377538
**WEE1**	0.551381703183312	**AP2M1**	0.0322382020495228	**ALAS1**	23.9091457589775	**EIF2B2**	0.144963120091208	JUN	2.27910640635946
**CLOCK**	0.541276442699399	**CDK2AP1**	0.0315960768790957	**MADD**	23.610217143035	**NFE2L2**	0.127090889959061	AHR	2.08641066658505
**ARNT**	0.510341236219266	**CRY1**	0.0260192637551128	**SCG5**	23.5584599878639	**PER3**	0.091787769071256	MAPK3	1.99897079303812
**PER2**	0.470238863055953	**HK1**	0.020820688745217	**CDK2AP1**	23.5469067680977	**DBP**	0.0907867845842636	ARNTL	1.95211068089632
**CCNA2**	0.439051786404152	**PCBD1**	0.0200202313409861	**PKM2**	23.4572802663874	**CRY1**	0.082970740931625	BHLHE40	1.94713716381873
**AHR**	0.416056158700292	**ALAS1**	0.0183753353564674	**CYP7B1**	23.4449074706404	**CRY2**	0.0756826260846772	PPARG	1.87394548486246
**PER3**	0.394401965276283	**PKM2**	0.0179531160663236	**GABARAPL1**	23.3945147175824	**PER2**	0.071064631749534	CEBPB	1.84665878053937
**JUN**	0.390762193059339	**ITGA5**	0.0178387650085763	**HK1**	23.1739586467746	**ADA**	0.0690243684292723	FOS	1.84102240012328
**CRY2**	0.389512630608661	**HIF1A**	0.017777191362097	**SLC27A1**	22.8176492281657	**NACA**	0.0657467403759448	NFIL3	1.7612499926888
**PER1**	0.385914004666131	**MADD**	0.0159827593789858	**EIF2B3**	22.5884323963263	**EIF2B3**	0.0615477588018198	AHCY	1.75501225622585
**HIF1A**	0.373867980001836	**PDGFRB**	0.0157804459691252	**CSNK1E**	22.5498678271054	**NPAS2**	0.0601731548069088	ITGA5	1.73523600034203
**FOS**	0.366660049188066	**EIF2B3**	0.0151383207986982	**CRY1**	22.5300456552822	**CCNA2**	0.0547892603398778	GSK3B	1.73339775429975
**CRY1**	0.343702809331051	**PRKCA**	0.0145929542155957	**SERPINE1**	22.4848488761108	**PER1**	0.0515484999929679	MYC	1.72885994647542
**TIMELESS**	0.305749308005903	**CYP7B1**	0.0142762897479879	**AHR**	22.3586129186158	**BNIP3**	0.0495391099313379	EGFR	1.71329372276415
**RXRA**	0.262643997630735	**CEBPB**	0.0135198135198135	**LGALS9**	22.252427091889	**HK1**	0.0452462291589491	CDKN1A	1.70167462718295
**CCNB1**	0.249481205055106	**SCG5**	0.013159167876149	**LDLR**	22.1424073916989	**ARNTL2**	0.0431626137068269	PDGFRB	1.67570618006341
**RARA**	0.212303842977224	**CSNK2A1**	0.0129216695254431	**SRF**	22.1323817719809	**FBXL3**	0.0427148551020932	NNMT	1.67192063943735
**EIF2B5**	0.211018680676354	**NR1D2**	0.0124202841183973	**SEC61G**	22.1049070311325	**CLOCK**	0.0395097804135942	AP2M1	1.64526410912342
**NPAS2**	0.205625544420401	**HEBP1**	0.0120684347099441	**CEBPB**	21.999792977691	**TIMELESS**	0.0393934593277985	PARP1	1.63582453346807
**CSNK2A1**	0.195935112886188	**AHR**	0.0118221401240269	**HSF1**	21.9210101165568	**BHLHE41**	0.0370993928249183	CDK1	1.54911430562063
**CREBBP**	0.190951965351357	**CSNK1E**	0.0117341777719136	**ITGA5**	21.8931454731799	**BHLHE40**	0.0359260940771708	VEGFA	1.48068779917142
**CSNK2B**	0.186506665124637	**TP53**	0.0113911245986718	**GLUL**	21.8821482612889	**ARNTL**	0.0343347266280929	HDAC3	1.47924179392207
**ITGA5**	0.186467727050273	**LDLR**	0.0112151998944452	**PDGFRB**	21.6377733649355	**CSNK2A2**	0.0294553443471268	AEBP1	1.46811686042305
**EIF2B3**	0.171980851648026	**IGFBP5**	0.0108985354268373	**HIF1A**	21.6135335177089	**CCRN4L**	0.0249820497668383	CCNA2	1.45373180796276

### Strongly connected components determination

**Figure 9:**
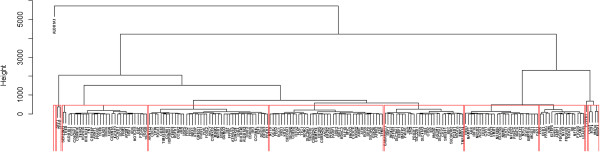
**Hierarchical clustering of the weighted interaction networks.** Genes are clustered according to the proximity of their expression profiles, evaluated by an Euclidean distance metrics.

After having scaled the individual fold changes with the closeness values of each gene, we have finally determined the collaborative actions of such genes by extracting the most cohesive clusters out of their interaction network. We performed a hierarchical clustering on the expression profiles of clock and clock controlled genes, by considering the Squared Euclidean distance as metric. We have subjected the found clusters to functional enrichment analysis against the GO FAT category, and determined five clusters significantly associated to at least a biological process or pathway (Figure 9).

**Cluster 1**, which is made of *CDK1, PER2, CCNA2, JUN, PER1, TIMELESS, CCNB1, CSNK2B, EIF2B3, NR1D1, ARNTL2, HSP90AA1, CSNK1D, E2F1, BHLHE40, CEBPB, TIPIN, ELAVL1, ATRIP, POLA1, MDM2, POLD1, HSPD1, HK1, LDHA, TRAF2, HSPA5, TBP, PPP5C, ALDH1A3, CCRN4L, CDC7, SUMO3, AHCY, POR, GNB2L1, ESRRA, ATP6V1D, USP5, DDX11, NRF1, FOXL2*, is strongly associated with several cellular processes: **cell cycle** (GO:0022402, p-value = 1.46E-02; GO:0000278, p-value = 2.40E-03; etc.), **DNA damage response activities** (GO:0000077, p-value = 1.055E-3; GO:0007050, p-value = 4.634E-3, etc.) and pathways, such as **cell cycle** (WikiPathways, WP179, p-value = 2.443E-4), and **hypoxia-inducible factor in the cardiovascular system** (BIOCARTA, p-value = 2.041E-2).

**Cluster 2**, which is made of *ARNTL, RXRA, CREBBP, ITGA5, SERPINE1, CCND1, CSNK1E, BHLHE41, CDKN1A, FN1, PKM2, PTGS2, DBP, PPARD, HSF1, THBD, IGFBP3, HSPA1A, AP2M1, TUBB, FASN, P4HA2, ALAS1, ALDH1B1, CDK2AP1, GYS1, NOTCH1, ADA, SLC7A8, SCG5*, is associated to the **inflammatory response** (GO:0006954, p-value = 3.043E-2), **response to lipid** (GO:0033993, p-value = 1.346E-3), **liver development** (GO:0001889, 1.025E-3) and **cell motility** (GO:0048870, p-value = 3.889E-3; GO:0016477, p-value = 1.823E-3).

**Cluster 3**, which is made of *WEE1, FOS, EGR1, PDGFRB, FOXO1, PPARG, IL6, NFYB, A2M, PDGFRA, PTGDS, ICAM1, IGFBP5, NFIL3, AEBP1, LGALS9*, is involved in the **response to cold** (GO:0009409, p-value = 3.126E-5) and **glucose homeostasis** (GO:0042593, p-value = 5.443E-3).

**Cluster 4**, which is made of *CLOCK, ARNT, PER3, EIF2B5, NPAS2, CSNK2A1, CSNK2A2, GSK3B, ATR, HDAC3, SIRT1, EIF2B2, PLAT, PARP1, HSF2, MYC, NFYA, RORA, CHEK2, ATM, NONO, KLF10, RBX1, TPMT, KITLG, TTC1, SEC61G, SEC61A1, GCLC, SLC29A1, RCC1, SLC9A8, APBB2, SRP9, CHMP1B, ENOX2, SLC9A9, ITFG2, WDR5, AACS*, cooperate with genes in cluster 1 to **negatively regulate cell cycle** (GO:0007050, p-value = 3.094E-3), to the **response to decreased oxygen levels** (GO:0036293, p-value = 3.128E-2) and to participate to the **P53 signaling pathway** (Pathway Ontology, PW:0000718, p-value = 1.466E-2).

**Cluster 5**, which is made of *AHR, HIF1A, CRY1, RB1, STAT1, NFKBIA, STAT5A, PRKCA, FBXL3, NR1D2, NFE2L2, HES1, ACSL1, CXCL12, NNMT, NAMPT, EGFR, FMR1, NPC1, PYGL, NACA, LDLR, GLUL, GLO1, MGST1, BMPR1A*, plays an important role in the positive regulation of **epithelial cell proliferation** (GO:0050678, p-value = 8.024E-7), **response to lipid** (GO:0033993, p-value = 3.898E-4; GO:0071396, p-value = 3.709E-3), **tissue morphogenesis** (GO:0048729, p-value = 7.487E-4) and takes part in the **adipogenesis pathway** (WikiPathways, WP236, p-value = 8.700E-4).

## Discussion

The molecular oscillator ticking in every cell regulates the customary sequence of intracellular activities, allowing the coordination of key pathways and the compartmentalization in the temporal dimension of poorly-compatible biochemical processes [40]. Altered functioning of the clock gene machinery may determine loss of time-of-day specific transcription of clock genes and clock controlled genes, causing severe deregulation of cellular homeostasis, cell dysfunction and biochemical and structural derangements that may lead to cell death and tissue dysfunction [41].

The aim of our study was to evaluate the functioning of the clock gene machinery in HS, and to address this issue we analyzed and compared mRNA expression levels of the core clock genes *CLOCK, NPAS2, ARNTL1, ARNTL2, PER1, PER2, PER3, CRY1, CRY2, CSNK1D, CSNK1E, CSNK2A1, CSNK2A2,CSNK2B, NR1D1*, *NR1D2*, *RORA*, *SIRT1, TIMELESS*, *TIPIN,* and a set of clock controlled genes in normal human fibroblasts and fibroblasts derived from subjects affected by Mucopolysaccharidosis type II. We also evaluated *in vitro* the effects of the treatment with idursulfase on circadian gene expression at different time points.

Analyzing data obtained from NGS we observed altered mRNA levels of some of the clock genes and clock controlled genes and a variable response to idursulfase treatment.

In HS fibroblasts *CLOCK* and *NPAS2* showed lower expression levels. *CLOCK* and its paralog *NPAS2* encode transcription factors belonging to the basic helix-loop-helix/Per-Arnt-Simpleminded (bHLH-PAS) domain family, and represent key elements in the positive limb of the transcriptional-translational feed-back loop that drives circadian rhythmicity of the oscillatory functions underlying cell homeostasis. In particular, their encoded proteins have histone acetyltransferase activity and are involved in chromatin remodeling with specificity for histones H3/H4 [42]. ARNTL, the heterodimerization partner of CLOCK and NPAS2, enhances their enzymatic function, and the decreased expression of these central components of the molecular clock could hinder the correct clock gene expression in the fibroblasts of Mucopolysaccharidosis type II patients.

A lower expression level was found in HS fibroblasts for *CRY1*, whereas *PER1* was up-regulated. The mammalian *Period* and *Cryptochrome* genes are both E-box-regulated genes, but in peripheral tissues *CRY1* mRNA expression peak is delayed by several hours with respect to that of *PER1*. *CRY1* usually shows evening-time expression and serving as a strong repressor at morning-time elements ensures a delay in feedback repression in the molecular clock. This phase delay in *CRY1* transcription is required for customary mammalian clock function [43]. The E-box (CACGTG) is crucial for daytime transcriptional activity and the delay originates from interactions between the proximal E-box and ROR response elements (RORE) present in the *CRY1* promoter [44].

A higher expression level was found in HS fibroblasts for *CSNK1D* and *CSNK1E*, encoding the serine/threonine protein kinase casein kinase (CK) I δ and ϵ respectively, which are key regulators of metazoan circadian rhythmicity. CKI binds to and phosphorylates the Period proteins, wich in turn interact with a variety of circadian regulators, suggesting the possibility that CKI may interact with and phosphorylate additional clock components as well [45]. Cryptochrome proteins are phosphorylated by CKI only when both proteins are bound to mammalian Period proteins [12]. ARNTL is also a substrate for CKI in vitro, and CKI activity positively regulates ARNTL-dependent transcription from circadian promoters in reporter assays [12]. CKI phosphorylates multiple circadian substrates and may exert its effects on circadian rhythm in part by a direct effect on ARNTL-dependent transcription [12,45]. The up-regulation of these protein kinases found in HS fibroblasts could play a crucial role in the derangement of the clock gene machinery and the downstream controlled pathways, but on the other hand CKs could represent important molecular targets for new therapeutic approaches.

In HS fibroblasts we found higher expression of *NR1D1*, and lower expression of *NR1D2*. *NR1D1* and *NR1D2*, activated by CLOCK/NPAS2:ARNTL/ARNTL2 heterodimers, code respectively for the orfan nuclear receptors REV ERBα and REV ERBβ, which realize a supplementary loop amplifying and stabilizing the oscillation of the molecular clockwork. REV-ERBα and REV-ERBβ act as negative transcriptional regulators by binding RORE in gene promoters, preventing the binding of the positive transcriptional regulator RORα, and negatively regulating the expression of *ARNTL*, *CLOCK*, and *CRY1*[46,47]. In addition, REV-ERBα is directly involved in lipid metabolism antagonizing the opposite role of RORα and inhibiting the expression of genes coding for apolipoprotein C-III, a constituent of very-low-density lipoproteins [48].

A lower expression level was found in HS fibroblasts for *SIRT1*, which regulates metabolic and stress responses acting in concert with the circadian rhythm machinery [49]. SIRT1 is involved in a number of cellular processes, including gene silencing at telomere and mating loci, and modulates cell survival by inhibiting apoptosis or cellular senescence induced by external challenges, including DNA damage and oxidative stress [50]. SIRT1 impinges on Ku70, peroxisome proliferator activated receptors, p53 and the forkhead box O (FOXO) family of transcription factors, and is modulated by active regulator of SIRT1 (AROS), hypermethylated in cancer 1 (HIC-1), deleted in breast cancer 1 (DBC1) and E2F transcription factor 1 (E2F1) [49-51]. Interestingly, *E2F1* was greatly over-expressed in HS fibroblasts, but normalized after 144 hours of IDS treatment, whereas *FOXO1* was severely down regulated, and responded faintly to IDS treatment. Besides, in HS fibroblasts there was severe down-regulation with no response to IDS treatment of *NAMPT*, encoding the enzyme controlling the synthesis of nicotinamide adenine dinucleotide (NAD^+^), a cofactor of SIRT1, linking nutrient sensing and circadian regulation [52].

After 24 hours of treatment with idursulfase in fibroblasts of patients with Mucopolysaccharidosis type II the expression evaluated by NGS of the genes *CLOCK*, *ARNTL2*, *NR1D1*, *NR1D2*, and *TIMELESS* showed higher expression levels compared to untreated HS fibroblasts. This effect faded completely after 144 hours, and at this time point *CSNK1E*, *CSNK2A1*, *CSNK2B*, *NR1D2*, and *TIMELESS* genes showed decreasing expression levels. Accordingly, the evaluation by qRT-PCR of clock gene expression upon synchronization through serum shock of normal fibroblasts and fibroblasts of patients affected by Hunter syndrome before and after 24 hours of idursulfase treatment evidenced a statistically significant effect on the expression levels of *ARNTL2*, *PER1* and *PER2*, but only at the earlier time points examined, with advance of the phase of oscillation of gene expression.

Regarding to the clock controlled genes, in Hunter syndrome fibroblasts there was alteration and dynamic change with idursulfase treatment at the time point considered of the expression of a huge number of output genes involved in the control of important cellular and tissue processes. An example is represented by *SERPINE1*, which showed higher expression levels in HS fibroblasts when compared to healthy fibroblasts, increased after 24 hours of idursulfase treatment, and decreased after 144 hours. *SERPINE1* codes for the plasminogen activator inhibitor (PAI)-1, the major component of inhibitors of fibrinolysis, whose activity shows a clear circadian oscillation peaking in the early morning. CLOCK:ARNTL and CLOCK:ARNTL2 heterodimers activate the *SERPINE1* promoter, driving the circadian variation in circulating PAI-1, and CLOCK:ARNTL2 heterodimer, binding two E-box enhancers in the promoter, shows double capability to activate PAI-1 expression [53].

The timing of processes that underlay cell function and the customary sequence of activation of key pathways depend on the correct ticking of the biological clock. Accordingly, the altered expression of clock controlled genes found in HS fibroblasts could hinder normal output of the clock gene machinery (*BHLHE40, BHLHE41, NFIL3*), and cellular activities, such as DNA transcription *(CTNNB1, FOS, FOXL2, FOXO1, HIF1A, HSF1, ID2, JUN, KEAP1, KITLG, KLF10, PPARA, PPARD, PPARG, RARA, RXRA, SMAD4, SP1, SREBF1, SRF, STAT1, STAT5A)*, post-translational modification and degradation (*FBXL3, SUMO3, USP5*), lipid and glucose metabolism and biosynthetic pathways (*ACSL1, AEBP1, ALAS1, APBB1, APBB1IP, APBB2, BMPR1A, CREBBP, FASN, GYS1, HMGCR, INSIG2, LPIN1, NAMPT*), molecular processing (*ADA, ALDH1A1, ALDH1A3, ALDH1B1, ATF2, CES1, CES2, CYP7B1, E2F1, EGFR, EGR1, EIF2B3, HSP90AA1, HSPA1A, HSPA5, HSPD1, GLO1, GLUL, HK1, LDHA, LMAN1, LMAN2, MAOA, NPC1, PARP1, PCK2, PRDX2, PYGL, SGMS2*), molecular transport (*AP2A1, AP2M1, IGFBP3, IGFBP5, LDLR, SLC22A15, SLC25A1, SLC27A1, SLC7A8, SLC9A3R2, SLC9A9*), DNA damage response (*ATM, ATR, ATRIP*), endoplasmic reticulum stress and unfolded protein response (*XBP1*), xenobiotic response (*AHR, ARNT*), autophagy (*BNIP3, CEBPB, GABARAPL1, ULK1*), cell cycle control (*CCNA2, CCNB1, CCND1, CDK2AP1, CDKN1A, GADD45A, MDM2, WEE1*), and tissue processes, such as inflammation, hemocoagulation and fibrinolysis (*ADAM17, A2M, FN1, ICAM1, IL6, ITGA5, MASP1, MGST1, NFKBIA, PDGFRA, PDGFRB, PTGS2, SERPINE1, SPP1, TFPI2, THBD, TRAF2,VEGFA*) [27-30,54].

The semantic hypergraph-based analysis of circadian gene expression brought out five gene clusters significantly associated to at least one biological process or pathway (cell cycle, DNA damage response, inflammation, liver development, cell motility, glucose homeostasis, response to cold and to decreased oxygen levels, the P53 signaling pathway, the positive regulation of epithelial cell proliferation, tissue morphogenesis, response to lipid, and the adipogenesis pathway). Besides, five genes, *AHR, HIF1A, CRY1, ITGA5*, and *EIF2B3,* were highlighted as top ranked by all the five considered topological indices and characterized by multifaceted centrality nature in the network analysis of the circadian transcriptome. The results suggest an interesting connection between circadian and hypoxic pathways and the toxicological signal mediator, the Aryl Hydrocarbon Receptor (AHR). Upon xenobiotic binding, AHR transcriptionally activates xenobiotic metabolizing enzymes and regulates molecules involved in the signaling of nuclear factor-erythroid 2-related factor-2 (Nrf2), p53 (TRP53), retinoblastoma (RB1), and NF-κB, influencing cellular responses to oxidative stress and inflammation. Through activation of these signaling pathways, regulation of cell cycle, and interactions with hypoxia-inducible factors (HIFs), AHR takes part in endogenous developmental functions during hematopoietic stem-cell maintenance and differentiation [55]. Intriguingly, the deficit of CRY proteins causes constitutive NF–κB and protein kinase A signaling activation, leading to unrelenting increase of proinflammatory cytokines, and *ITGA5* encodes a receptor for fibronectin and fibrinogen, playing a role in chemotaxis, leukocyte migration, cell-cell adhesion, angiogenesis, and blood coagulation [56,57].

On the other hand, a cross-talk exists between circadian and AHR signaling pathways at genetic, epigenetic and proteomic level, and this interaction might play a role in the regulatory influences maneuvered by circadian rhythmicity on cell physiology [58]. Regarding to the interaction between hypoxic pathways and AHR complex, it has been proven that in endothelial cells AHR has a physiological function in the absence of exogenous ligands, and upon activation mediates toxicity of halogenated aromatic hydrocarbons. In addition, evidence suggests that hypoxia induces HIF dependent gene expression, significantly reducing AHR expression level and AHR-mediated expression of cytochrome P-450 phase I xenobiotic metabolizing enzyme CYP1A1 [59]. A cross-talk exists between hypoxic and circadian pathways operated by the PAS protein family members PER and CLOCK and HIF-1α. Interestingly, the components of AHR, circadian and hypoxic pathways are characterized by a PAS domain that serves as an interface for protein-protein interactions [60]. Moreover, cell proliferation and differentiation both *in vitro* and *in vivo* are highly influenced by oxygen concentration, and neuronal stem cell proliferation and neuronal and oligodendroglial differentiation are enhanced by a mild level of hypoxia [61]. Intriguingly, *EIF2B3* encodes one of the subunits of initiation factor EIF2B, which catalyzes the exchange of eukaryotic initiation factor 2-bound guanosine-diphosphate (GDP) for guanosine-5'-triphosphate (GTP), representing an essential factor for protein synthesis, and mutations in this gene have been associated with neurodegenerative and white matter diseases [62-64].

Taking into account some limitations of the study, represented by the limited number of patients, suffering from a disease that is clinically heterogeneous in terms of onset, severity and progression, the dynamic variation of expression levels observed for core clock genes and clock controlled genes in the fibroblasts of patients affected by HS at different time points of treatment with idursulfase may be an indirect evidence of the key role played by the molecular clock in the regulation of the complex array of cellular functions in this mucopolysaccharidosis. A paradigm is represented by the altered expression of the clock controlled genes *NPC1* and *SGMS2*, which are down-regulated in HS fibroblasts and increase temporarily after idursulfase treatment.

## Conclusion

Biological processes show time related variations driven by genetically encoded oscillators operated by transcriptional/translational feedback loops hardwired by the clock genes and their coded circadian proteins. Data obtained in our study show that circadian gene expression is variably altered in the fibroblasts of subjects affected by HS, the treatment with idursulfase determines some modifications in the expression levels of clock genes and clock controlled genes, but these changes are temporary and fade in the course of treatment. Altered functioning of the clock gene machinery in HS may determine severe deregulation of cellular homeostasis, cell dysfunction and biochemical and structural derangements that may lead to cell death. These alterations are related to the key role played by the molecular clockwork in the control of downstream gene expression regulating a complex array of cellular functions, such as molecule biosynthesis, post-translational modification, processing, transport, conjugation, internalization and degradation, and cell processes such as cell cycle, autophagy, apoptosis and DNA damage response.The alteration of clock gene expression levels and the response to the deficient enzyme suggest a direct involvement of the molecular clock machinery in the physiopathology of cellular derangements that characterize Mucopolysaccharidosis type II, opening the way to possible new therapeutic strategies.

## Competing interest

The authors declare that there are no conflicts of interest with respect to the authorship and/or publication of this article.

## Authors’ contributions

GM and M Scarpa conceived the study, participated in its design and coordination and helped to draft the manuscript; RT, FD, AZ and VP carried out cell cultures and molecular genetic studies; M Salvalaio and LR participated in experimental plan design and whole transcriptome data production/analysis, MV participated in the study coordination and helped to draft the manuscript, MF, FG and TM performed bioinformatic and statistical analyses. All authors read and approved the final manuscript.

## Pre-publication history

The pre-publication history for this paper can be accessed here:

http://www.biomedcentral.com/1755-8794/6/37/prepub

## Supplementary Material

Additional file 1: Table S1 Expression levels of core clock genes and clock controlled genes evaluated by whole transcriptome analysis performed through Next Generation Sequencing technology in normal human fibroblasts (Control) and fibroblasts of mucopolysaccharidosis Type II patients before (Hunter), 24 hours (T1) and 144 hours (T2) after idursulfase treatment. Click here for file

## References

[B1] Lampe C Bellettato CM Karabul N Scarpa M Mucopolysaccharidoses and other lysosomal storage diseases Rheum Dis Clin North Am 2013 39 431 455 10.1016/j.rdc.2013.03.004 23597973

[B2] Scarpa M Almássy Z Beck M Bodamer O Bruce IA De Meirleir L Guffon N Guillén-Navarro E Hensman P Jones S Kamin W Kampmann C Lampe C Lavery CA Teles EL Link B Lund AM Malm G Pitz S Rothera M Stewart C Tylki-Szymańska A van der Ploeg A Walker R Zeman J Wraith JE Hunter Syndrome European Expert Council: Mucopolysaccharidosis type II: European recommendations for the diagnosis and multidisciplinary management of a rare disease Orphanet J Rare Dis 2011 6 72 10.1186/1750-1172-6-72 22059643 PMC3223498

[B3] Lowrey PL Takahashi JS Genetics of the mammalian circadian system: photic entrainment, circadian pacemaker mechanisms, and posttranslational regulation Annu Rev Genet 2000 34 533 562 10.1146/annurev.genet.34.1.533 11092838

[B4] Mazzoccoli G The timing clockwork of life J Biol Regul Homeost Agents 2011 25 137 143 21382283

[B5] Bass J Circadian topology of metabolism Nature 2012 491 348 356 10.1038/nature11704 23151577

[B6] Schibler U Sassone-Corsi P A web of circadian pacemakers Cell 2002 111 919 922 10.1016/S0092-8674(02)01225-4 12507418

[B7] Reyes BA Pendergast JS Yamazaki S Mammalian peripheral circadian oscillators are temperature compensated J Biol Rhythms 2008 23 95 98 10.1177/0748730407311855 18258762 PMC2365757

[B8] Hastings MH Reddy AB Maywood ES A clockwork web: circadian timing in brain and periphery, in health and disease Nat Rev Neurosci 2003 4 649 661 12894240 10.1038/nrn1177

[B9] Houben T Deboer T van Oosterhout F Meijer JH Correlation with behavioral activity and rest implies circadian regulation by SCN neuronal activity levels J Biol Rhythms 2009 24 477 487 10.1177/0748730409349895 19926807

[B10] Pezuk P Mohawk JA Yoshikawa T Sellix MT Menaker M Circadian organization is governed by extra-SCN pacemakers J Biol Rhythms 2010 25 432 441 10.1177/0748730410385204 21135159

[B11] Nagoshi E Saini C Bauer C Laroche T Naef F Schibler U Circadian gene expression in individual fibroblasts: cell-autonomous and self-sustained oscillators pass time to daughter cells Cell 2004 119 693 705 10.1016/j.cell.2004.11.015 15550250

[B12] Eide EJ Vielhaber EL Hinz WA Virshup DM The circadian regulatory proteins BMAL1 and Cryptochromes are substrates of Casein Kinase Iϵ J Biol Chem 2002 277 17248 17254 10.1074/jbc.M111466200 11875063 PMC1513548

[B13] Cho H Zhao X Hatori M Yu RT Barish GD Lam MT Chong LW DiTacchio L Atkins AR Glass CK Liddle C Auwerx J Downes M Panda S Evans RM Regulation of circadian behaviour and metabolism by REV-ERB-α and REV-ERB-β Nature 2012 485 123 127 10.1038/nature11048 22460952 PMC3367514

[B14] Mazzoccoli G Cai Y Liu S Francavilla M Giuliani F Piepoli A Pazienza V Vinciguerra M Yamamoto T Takumi T REV-ERBα and the clock gene machinery in mouse peripheral tissues: a possible role as a synchronizing hinge J Biol Regul Homeost Agents 2012 26 265 276 22824754

[B15] Asher G Schibler U Crosstalk between components of circadian and metabolic cycles in mammals Cell Metab 2011 13 125 137 10.1016/j.cmet.2011.01.006 21284980

[B16] Unsal-Kaçmaz K Chastain PD Qu PP Minoo P Cordeiro-Stone M Sancar A Kaufmann WK The human Tim/Tipin complex coordinates an Intra-S checkpoint response to UV that slows replication fork displacement Mol Cell Biol 2007 27 3131 3142 10.1128/MCB.02190-06 17296725 PMC1899931

[B17] Smith KD Fu MA Brown EJ Tim-Tipin dysfunction creates an indispensible reliance on the ATR-Chk1 pathway for continued DNA synthesis J Cell Biol 2009 5 15 23 19805627 10.1083/jcb.200905006 PMC2762102

[B18] Yang X Wood PA Hrushesky WJ Mammalian TIMELESS is required for ATM-dependent CHK2 activation and G2/M checkpoint control J Biol Chem 2010 285 3030 3034 10.1074/jbc.M109.050237 19996108 PMC2823431

[B19] Kemp MG Akan Z Yilmaz S Grillo M Smith-Roe SL Kang TH Cordeiro-Stone M Kaufmann WK Abraham RT Sancar A Unsal-Kaçmaz K Tipin-replication protein A interaction mediates Chk1 phosphorylation by ATR in response to genotoxic stress J Biol Chem 2010 285 16562 16571 10.1074/jbc.M110.110304 20233725 PMC2878033

[B20] Matsuo T Yamaguchi S Mitsui S Emi A Shimoda F Okamura H Control mechanism of the circadian clock for timing of cell division in vivo Science 2003 302 255 259 10.1126/science.1086271 12934012

[B21] Filipski E King VM Etienne MC Li XM Claustrat B Granda TG Persistent twenty-four hour changes in liver and bone marrow despite suprachiasmatic nuclei ablation in mice Am J Physiol Regul Integr Comp Physiol 2004 287 R844 R851 10.1152/ajpregu.00085.2004 15217787

[B22] Hunt T Sassone-Corsi P Riding tandem: circadian clocks and the cell cycle Cell 2007 129 461 464 10.1016/j.cell.2007.04.015 17482541

[B23] Ma D Panda S Lin JD Temporal orchestration of circadian autophagy rhythm by C/EBPβ EMBO J 2011 30 4642 4651 10.1038/emboj.2011.322 21897364 PMC3243590

[B24] Mazzoccoli G Sothern RB Greco A Pazienza V Vinciguerra M Liu S Cai Y Time-related dynamics of variation in core clock gene expression levels in tissues relevant to the immune system Int J Immunopathol Pharmacol 2011 24 869 879 22230394 10.1177/039463201102400406

[B25] Vinciguerra M Borghesan M Pazienza V Piepoli A Palmieri O Tarquini R Tevy MF De Cata A Mazzoccoli G The transcriptional regulators, the circadian clock and the immune system J Biol Regul Homeost Agents 2013 27 9 22 23489683

[B26] Tevy MF Giebultowicz J Pincus Z Mazzoccoli G Vinciguerra M Aging signaling pathways and circadian clock-dependent metabolic derangements Trends Endocrinol Metab 2013 24 229 237 10.1016/j.tem.2012.12.002 23299029 PMC3624052

[B27] Panda S Antoch MP Miller BH Su AI Schook AB Straume M Schultz PG Kay SA Takahashi JS Hogenesch JB Coordinated transcription of key pathways in the mouse by the circadian clock Cell 2002 109 307 320 10.1016/S0092-8674(02)00722-5 12015981

[B28] Hughes ME DiTacchio L Hayes KR Vollmers C Pulivarthy S Baggs JE Panda S Hogenesch JB Harmonics of circadian gene transcription in mammals PLoS Genet 2009 5 e1000442 10.1371/journal.pgen.1000442 19343201 PMC2654964

[B29] Bozek K Relógio A Kielbasa SM Heine M Dame C Kramer A Herzel H Regulation of clock-controlled genes in mammals PLoS One 2009 4 e4882 10.1371/journal.pone.0004882 19287494 PMC2654074

[B30] Sukumaran S Almon RR DuBois DC Jusko WJ Circadian rhythms in gene expression: Relationship to physiology, disease, drug disposition and drug action Adv Drug Deliv Rev 2010 62 904 917 10.1016/j.addr.2010.05.009 20542067 PMC2922481

[B31] Carstea ED Morris JA Coleman KG Loftus SK Zhang D Cummings C Gu J Rosenfeld MA Pavan WJ Krizman DB Nagle J Polymeropoulos MH Sturley SL Ioannou YA Higgins ME Comly M Cooney A Brown A Kaneski CR Blanchette-Mackie EJ Dwyer NK Neufeld EB Chang TY Liscum L Strauss JF 3rd Ohno K Zeigler M Carmi R Sokol J Markie D O'Neill RR van Diggelen OP Elleder M Patterson MC Brady RO Vanier MT Pentchev PG Tagle DA Niemann-Pick C1 disease gene: homology to mediators of cholesterol homeostasis Science 1997 277 228 231 10.1126/science.277.5323.228 9211849

[B32] Harzer K Massenkeil G Frohlich E Concurrent increase of cholesterol, sphingomyelin and glucosylceramide in the spleen from non-neurologic Niemann-Pick type C patients but also patients possibly affected with other lipid trafficking disorders FEBS Lett 2003 537 177 181 10.1016/S0014-5793(03)00100-5 12606053

[B33] Pizarro A Hayer K Lahens NF Hogenesch JB CircaDB: a database of mammalian circadian gene expression profiles Nucleic Acids Res 2013 41 D1009 D1013 10.1093/nar/gks1161 23180795 PMC3531170

[B34] Balsalobre A Damiola F Schibler U A serum shock induces circadian gene expression in mammalian tissue culture cells Cell 1998 93 929 937 10.1016/S0092-8674(00)81199-X 9635423

[B35] Castellana S Mazza T Congruency in the prediction of pathogenic missense mutations: state-of-the-art web-based tools Brief Bioinform 2013 [Epub ahead of print] 10.1093/bib/bbt013 23505257

[B36] Castellana S Romani M Valente EM Mazza T A solid quality-control analysis of AB SOLiD short-read sequencing data Brief Bioinform 2012 [Epub ahead of print] 10.1093/bib/bbs048 22877770

[B37] Wasserman S Faust K Social Network Analysis: Methods and Applications 1994 Cambridge, UK: Cambridge University Press

[B38] Mazza T Romanel A Jordán J Estimating the divisibility of complex biological networks by sparseness indices Brief Bioinformatics 2010 11 364 374 10.1093/bib/bbp060 20064873

[B39] Mazza T Ballarini P Guido R Prandi D The relevance of topology in parallel simulation of biological networks IEEE/ACM Trans Comput Biol Bioinform (TCBB) 2012 9 911 923 10.1109/TCBB.2012.27 22331861

[B40] Mazzoccoli G Pazienza V Vinciguerra M Clock genes and clock controlled genes in the regulation of metabolic rhythms Chronobiol Int 2012 29 227 251 10.3109/07420528.2012.658127 22390237

[B41] Kondratov RV Antoch MP Circadian proteins in the regulation of cell cycle and genotoxic stress responses Trends Cell Biol 2007 17 311 317 10.1016/j.tcb.2007.07.001 17644383

[B42] Doi M Hirayama J Sassone-Corsi P Circadian regulator CLOCK is a histone acetyltransferase Cell 2006 125 497 508 10.1016/j.cell.2006.03.033 16678094

[B43] Ukai-Tadenuma M Yamada RG Xu H Ripperger JA Liu AC Ueda HR Delay in feedback repression by cryptochrome 1 is required for circadian clock function Cell 2011 144 268 281 10.1016/j.cell.2010.12.019 21236481

[B44] Fustin JM O'Neill JS Hastings MH Hazlerigg DG Dardente H Cry1 circadian phase in vitro: wrapped up with an E-box J Biol Rhythms 2009 24 16 24 10.1177/0748730408329267 19150926

[B45] Agostino PV Harrington ME Ralph MR Golombek DA Casein kinase-1-epsilon (CK1epsilon) and circadian photic responses in hamsters Chronobiol Int 2009 26 126 133 10.1080/07420520802675177 19142762

[B46] Burris TP Nuclear hormone receptors for heme: REV-ERBalpha and REV-ERBbeta are ligand-regulated components of the mammalian clock Mol Endocrinol 2008 22 1509 1520 10.1210/me.2007-0519 18218725 PMC5419435

[B47] Tahara Y Otsuka M Fuse Y Hirao A Shibata S Refeeding after fasting elicits insulin-dependent regulation of Per2 and Rev-erbα with shifts in the liver clock J Biol Rhythms 2011 26 230 240 10.1177/0748730411405958 21628550

[B48] Raspe’ E Duez H Mansen A Fontaine C Fievet C Fruchart JC Vennstrom B Staels B Identification of Rev-erb alpha as a physiological repressor of apoC-III gene transcription J Lipid Res 2002 43 2172 2179 10.1194/jlr.M200386-JLR200 12454280

[B49] Asher G Gatfield D Stratmann M Reinke H Dibner C Kreppel F Mostoslavsky R Alt FW Schibler U SIRT1 regulates circadian clock gene expression through PER2 Deacetylation Cell 2008 134 317 328 10.1016/j.cell.2008.06.050 18662546

[B50] Guarente L Franklin H Epstein lecture: Sirtuins, aging, and medicine N Engl J Med 2011 364 2235 2244 10.1056/NEJMra1100831 21651395

[B51] Zhao W Kruse JP Tang Y Jung SY Qin J Gu W Negative regulation of the deacetylase SIRT1 by DBC1 Nature 2008 451 587 590 10.1038/nature06515 18235502 PMC2866287

[B52] Brooks CL Gu W How does SIRT1 affect metabolism, senescence and cancer? Nat Rev Cancer 2009 9 123 128 10.1038/nrc2562 19132007 PMC2857763

[B53] Schoenhard JA Smith LH Painter CA Eren M Johnson CH Vaughan DE Regulation of the PAI-1 promoter by circadian clock components: differential activation by BMAL1 and BMAL2 J Mol Cell Cardiol 2003 35 473 481 10.1016/S0022-2828(03)00051-8 12738229

[B54] Cermakian N Lange T Golombek D Sarkar D Nakao A Shibata S Mazzoccoli G Crosstalk between the circadian clock circuitry and the immune system Chronobiol Int 2013 1 19 DOI: 10.3109/07420528.2013.782315 23697902 10.3109/07420528.2013.782315 PMC7195843

[B55] Lindsey S Papoutsakis ET The evolving role of the aryl hydrocarbon receptor (AHR) in the normophysiology of hematopoiesis Stem Cell Rev 2012 8 1223 1235 10.1007/s12015-012-9384-5 22628113 PMC3463730

[B56] Narasimamurthy R Hatori M Nayak SK Liu F Panda S Verma IM Circadian clock protein cryptochrome regulates the expression of proinflammatory cytokines Proc Natl Acad Sci USA 2012 109 12662 12667 10.1073/pnas.1209965109 22778400 PMC3411996

[B57] Chu TJ Peters DG Serial analysis of the vascular endothelial transcriptome under static and shear stress conditions Physiol Genomics 2008 34 185 192 10.1152/physiolgenomics.90201.2008 18505769

[B58] Anderson G Beischlag TV Vinciguerra M Mazzoccoli G The circadian clock circuitry and the AHR signaling pathway in physiology and pathology Biochem Pharmacol 2013 doi:pii: S0006-2952(13)00126-3. 10.1016/j.bcp.2013.02.022. [Epub ahead of print] 10.1016/j.bcp.2013.02.022 23438471

[B59] Zhang N Walker MK Crosstalk between the aryl hydrocarbon receptor and hypoxia on the constitutive expression of cytochromeP4501A1 mRNA Cardiovasc Toxicol 2007 7 282 290 10.1007/s12012-007-9007-6 17968679 PMC2213366

[B60] Chilov D Hofer T Bauer C Wenger RH Gassmann M Hypoxia affects expression of circadian genes PER1 and CLOCK in mouse brain FASEB J 2001 15 2613 2622 10.1096/fj.01-0092com 11726537

[B61] Santilli G Lamorte G Carlessi L Ferrari D Rota Nodari L Binda E Delia D Vescovi AL De Filippis L Mild hypoxia enhances proliferation and multipotency of human neural stem cells PLoS One 2010 5 e8575 10.1371/journal.pone.0008575 20052410 PMC2797394

[B62] Oliveira SA Li YJ Noureddine MA Zuchner S Qin X Pericak-Vance MA Vance JM Identification of risk and age-at-onset genes on chromosome 1p in Parkinson disease Am J Hum Genet 2005 77 252 264 10.1086/432588 15986317 PMC1224528

[B63] Ohlenbusch A Henneke M Brockmann K Goerg M Hanefeld F Kohlschütter A Gärtner J Identification of ten novel mutations in patients with eIF2B-related disorders Hum Mutat 2005 25 411 15776425 10.1002/humu.9325

[B64] Scali O Di Perri C Federico A The spectrum of mutations for the diagnosis of vanishing white matter disease Neurol Sci 2006 27 271 277 10.1007/s10072-006-0683-y 16998732

