# Supplementary material for: Circadian transcriptome analysis in human fibroblasts from Hunter syndrome and impact of iduronate-2-sulfatase treatment
Source: BMC Med Genomics. 2013 Oct 2;6:37. doi: 10.1186/1755-8794-6-37 (PMC3851237; doi:10.1186/1755-8794-6-37)
Supplement: Additional file 1: Table S1 — Expression levels of core clock genes and clock controlled genes evaluated by whole transcriptome analysis performed through Next Generation Sequencing technology in normal human fibroblasts (Control) and fibroblasts of mucopolysaccharidosis Type II patients before (Hunter), 24 hours (T1) and 144 hours (T2) after idursulfase treatment. [file 1755-8794-6-37-S1.doc]

**Additional file 1: Table S1. Expression levels of core clock genes and clock controlled genes evaluated by whole transcriptome analysis performed through Next Generation Sequencing technology in normal human fibroblasts (Control) and fibroblasts of mucopolysaccharidosis Type II patients before (Hunter), 24 hours (T1) and 144 hours (T2) after idursulfase treatment**

|  |  | **HUNTER/CONTROL** | | | | **T1/CONTROL** | | | | **T2/CONTROL** | | | | **T1/HUNTER** | | | | **T2/HUNTER** | | | |
| --- | --- | --- | --- | --- | --- | --- | --- | --- | --- | --- | --- | --- | --- | --- | --- | --- | --- | --- | --- | --- | --- |
| **CORE CLOCK GENES** | **Gene Names** | **ratio** | **log2 (ratio)** | **fold change** | **p** | **ratio** | **log2 (ratio)** | **fold change** | **p** | **ratio** | **log2 (ratio)** | **fold change** | **p** | **ratio** | **log2 (ratio)** | **fold change** | **p** | **ratio** | **log2 (ratio)** | **fold change** | **p** |
| Bmal1 (Arntl, MOP3) | **ARNTL** | 1.344 | 0.427 | 1.344 | 0.014 | 1.535 | 0.618 | 1.535 | <0.001 | 1.387 | 0.472 | 1.387 | 0.008 | 1.142 | 0.191 | 1.142 | 0.215 | 1.032 | 0.045 | 1.032 | 0.783 |
| Bmal2 (Arntl2, MOP9) | **ARNTL2** | 1.093 | 0.128 | 1.093 | 0.472 | 2.220 | 1.151 | 2.220 | <0.001 | 0.737 | -0.440 | -1.356 | 0.031 | 2.031 | 1.023 | 2.031 | <0.001 | 0.674 | -0.568 | -1.483 | 0.004 |
| Clock | **CLOCK** | 0.689 | -0.537 | -1.451 | <0.001 | 0.881 | -0.183 | -1.135 | 0.003 | 0.601 | -0.735 | -1.664 | <0.001 | 1.278 | 0.354 | 1.278 | <0.001 | 0.872 | -0.198 | -1.147 | 0.010 |
| Cryptochrome 1 (Cry1) | **CRY1** | 0.661 | -0.597 | -1.513 | <0.001 | 0.570 | -0.812 | -1.756 | <0.001 | 0.498 | -1.007 | -2.010 | <0.001 | 0.862 | -0.215 | -1.160 | 0.086 | 0.753 | -0.409 | -1.328 | 0.002 |
| Cryptochrome 2 (Cry2) | **CRY2** | 1.168 | 0.224 | 1.168 | 0.029 | 1.044 | 0.062 | 1.044 | 0.559 | 1.054 | 0.076 | 1.054 | 0.487 | 0.893 | -0.163 | -1.119 | 0.106 | 0.902 | -0.149 | -1.109 | 0.152 |
| Caseinkinase 1 δ | **CSNK1D** | 1.266 | 0.340 | 1.266 | <0.001 | 1.127 | 0.173 | 1.127 | 0.007 | 1.119 | 0.162 | 1.119 | 0.015 | 0.891 | -0.167 | -1.123 | 0.005 | 0.884 | -0.178 | -1.132 | 0.004 |
| Caseinkinase 1 ε | **CSNK1E** | 1.543 | 0.625 | 1.543 | <0.001 | 1.469 | 0.555 | 1.469 | <0.001 | 1.274 | 0.349 | 1.274 | <0.001 | 0.952 | -0.070 | -1.050 | 0.191 | 0.826 | -0.276 | -1.211 | <0.001 |
| Casein Kinase 2 α 1 Polypeptide | **CSNK2A1** | 1.024 | 0.035 | 1.024 | 0.562 | 1.043 | 0.061 | 1.043 | 0.309 | 0.873 | -0.196 | -1.146 | 0.002 | 1.018 | 0.026 | 1.018 | 0.658 | 0.852 | -0.231 | -1.174 | <0.001 |
| Casein Kinase 2 α 2 Polypeptide | **CSNK2A2** | 0.917 | -0.124 | -1.090 | 0.261 | 0.916 | -0.127 | -1.092 | 0.252 | 0.777 | -0.364 | -1.287 | 0.002 | 0.998 | -0.002 | -1.002 | 0.983 | 0.847 | -0.239 | -1.180 | 0.048 |
| Casein Kinase 2 β Polypeptide | **CSNK2B** | 1.222 | 0.290 | 1.222 | <0.001 | 1.152 | 0.205 | 1.152 | <0.001 | 1.003 | 0.004 | 1.003 | 0.856 | 0.943 | -0.085 | -1.061 | <0.001 | 0.820 | -0.285 | -1.219 | <0.001 |
| Npas2 | **NPAS2** | 0.604 | -0.728 | -1.657 | <0.001 | 0.717 | -0.481 | -1.395 | 0.001 | 0.637 | -0.651 | -1.570 | <0.001 | 1.187 | 0.248 | 1.187 | 0.113 | 1.055 | 0.078 | 1.055 | 0.641 |
| Rev Erb α | **NR1D1** | 1.994 | 0.995 | 1.994 | <0.001 | 3.599 | 1.848 | 3.599 | <0.001 | 1.719 | 0.782 | 1.719 | <0.001 | 1.805 | 0.852 | 1.805 | <0.001 | 0.862 | -0.214 | -1.160 | 0.014 |
| Rev Erb β | **NR1D2** | 0.506 | -0.982 | -1.975 | <0.001 | 0.604 | -0.727 | -1.655 | <0.001 | 0.394 | -1.343 | -2.537 | <0.001 | 1.193 | 0.255 | 1.193 | <0.001 | 0.778 | -0.361 | -1.285 | <0.001 |
| Period 1 (Per1) | **PER1** | 1.278 | 0.354 | 1.278 | <0.001 | 1.223 | 0.291 | 1.223 | 0.004 | 1.029 | 0.042 | 1.029 | 0.704 | 0.957 | -0.063 | -1.045 | 0.505 | 0.805 | -0.312 | -1.242 | 0.002 |
| Period 2 (Per2) | **PER2** | 1.402 | 0.488 | 1.402 | 0.001 | 1.729 | 0.790 | 1.729 | <0.001 | 1.202 | 0.266 | 1.202 | 0.104 | 1.233 | 0.302 | 1.233 | 0.022 | 0.857 | -0.222 | -1.166 | 0.138 |
| Period 3 (Per3) | **PER3** | 0.983 | -0.025 | -1.018 | 0.794 | 1.145 | 0.195 | 1.145 | 0.037 | 0.922 | -0.117 | -1.084 | 0.253 | 1.165 | 0.220 | 1.165 | 0.018 | 0.939 | -0.091 | -1.065 | 0.369 |
| Ror α | **RORA** | 0.934 | -0.099 | -1.071 | 0.287 | 0.872 | -0.197 | -1.146 | 0.037 | 0.779 | -0.360 | -1.284 | <0.001 | 0.934 | -0.098 | -1.070 | 0.301 | 0.834 | -0.262 | -1.199 | 0.010 |
| Sirtuin-1 | **SIRT1** | 0.764 | -0.389 | -1.309 | <0.001 | 0.887 | -0.174 | -1.128 | 0.074 | 0.708 | -0.499 | -1.413 | <0.001 | 1.161 | 0.215 | 1.161 | 0.037 | 0.927 | -0.110 | -1.079 | 0.330 |
| Timeless | **TIMELESS** | 1.257 | 0.330 | 1.257 | 0.006 | 2.414 | 1.271 | 2.414 | <0.001 | 0.518 | -0.950 | -1.932 | <0.001 | 1.919 | 0.941 | 1.919 | <0.001 | 0.412 | -1.281 | -2.430 | <0.001 |
| Timeless Interacting Protein | **TIPIN** | 0.797 | -0.328 | -1.255 | 0.274 | 1.462 | 0.548 | 1.462 | 0.034 | 0.607 | -0.720 | -1.647 | 0.031 | 1.836 | 0.876 | 1.836 | 0.001 | 0.762 | -0.392 | -1.312 | 0.262 |
| **CLOCK CONTROLLED GENES** | **Gene Names** | **ratio** | **log2 (ratio)** | **fold change** | **p** | **ratio** | **log2 (ratio)** | **fold change** | **p** | **ratio** | **log2 (ratio)** | **fold change** | **p** | **ratio** | **log2 (ratio)** | **fold change** | **p** | **ratio** | **log2 (ratio)** | **fold change** | **p** |
| Alpha-2-Macroglobulin | **A2M** | 0.220 | -2.182 | -4.539 | <0.001 | 0.177 | -2.497 | -5.645 | <0.001 | 0.243 | -2.039 | -4.110 | <0.001 | 0.804 | -0.315 | -1.244 | 0.018 | 1.104 | 0.143 | 1.104 | 0.259 |
| Acetoacetyl-Coa Synthetase | **AACS** | 0.908 | -0.139 | -1.101 | 0.125 | 1.041 | 0.057 | 1.041 | 0.512 | 0.850 | -0.234 | -1.176 | 0.014 | 1.146 | 0.196 | 1.146 | 0.027 | 0.936 | -0.095 | -1.068 | 0.328 |
| Acyl-Coa Dehydrogenase. C-2 To C-3 Short Chain | **ACADS** | 1.021 | 0.030 | 1.021 | 0.900 | 1.157 | 0.210 | 1.157 | 0.361 | 0.845 | -0.243 | -1.184 | 0.345 | 1.133 | 0.180 | 1.133 | 0.427 | 0.827 | -0.273 | -1.209 | 0.282 |
| Acyl-Coa Synthetase Long-Chain Family Member 1 | **ACSL1** | 0.490 | -1.028 | -2.039 | <0.001 | 0.681 | -0.554 | -1.468 | <0.001 | 0.399 | -1.327 | -2.509 | <0.001 | 1.389 | 0.474 | 1.389 | <0.001 | 0.813 | -0.299 | -1.231 | 0.001 |
| Adenosine Deaminase | **ADA** | 2.464 | 1.301 | 2.464 | <0.001 | 2.741 | 1.455 | 2.741 | <0.001 | 1.878 | 0.909 | 1.878 | <0.001 | 1.113 | 0.154 | 1.113 | 0.368 | 0.762 | -0.392 | -1.312 | 0.044 |
| ADAM Metallopeptidase Domain 17 | **ADAM17** | 0.604 | -0.728 | -1.656 | <0.001 | 0.622 | -0.685 | -1.608 | <0.001 | 0.533 | -0.909 | -1.878 | <0.001 | 1.030 | 0.042 | 1.030 | 0.682 | 0.882 | -0.182 | -1.134 | 0.101 |
| AE Binding Protein 1 | **AEBP1** | 0.501 | -0.996 | -1.995 | <0.001 | 0.436 | -1.199 | -2.296 | <0.001 | 0.440 | -1.185 | -2.274 | <0.001 | 0.869 | -0.203 | -1.151 | <0.001 | 0.877 | -0.189 | -1.140 | <0.001 |
| Adenosylhomocysteinase | **AHCY** | 1.029 | 0.041 | 1.029 | 0.467 | 1.283 | 0.360 | 1.283 | <0.001 | 1.414 | 0.500 | 1.414 | <0.001 | 1.248 | 0.319 | 1.248 | <0.001 | 1.375 | 0.459 | 1.375 | <0.001 |
| Aryl Hydrocarbon Receptor | **AHR** | 0.688 | -0.539 | -1.453 | <0.001 | 0.592 | -0.755 | -1.688 | <0.001 | 0.611 | -0.711 | -1.637 | <0.001 | 0.861 | -0.216 | -1.162 | <0.001 | 0.888 | -0.172 | -1.127 | 0.002 |
| Aminolevulinate. Delta-. Synthase 1 | **ALAS1** | 1.246 | 0.318 | 1.246 | 0.001 | 1.590 | 0.669 | 1.590 | <0.001 | 1.175 | 0.233 | 1.175 | 0.018 | 1.276 | 0.352 | 1.276 | <0.001 | 0.943 | -0.085 | -1.061 | 0.359 |
| Aldehyde Dehydrogenase 1 Family. Member A1 | **ALDH1A1** | 0.019 | -5.696 | -51.836 | <0.001 | 0.004 | -7.817 | -225.517 | <0.001 | 0.007 | -7.198 | -146.865 | <0.001 | 0.230 | -2.121 | -4.351 | <0.001 | 0.353 | -1.502 | -2.833 | <0.001 |
| Aldehyde Dehydrogenase 1 Family. Member A3 | **ALDH1A3** | 2.000 | 1.000 | 2.000 | <0.001 | 1.121 | 0.165 | 1.121 | 0.025 | 0.992 | -0.012 | -1.008 | 0.883 | 0.561 | -0.835 | -1.783 | <0.001 | 0.496 | -1.012 | -2.016 | <0.001 |
| Aldehyde Dehydrogenase 1 Family. Member B1 | **ALDH1B1** | 1.996 | 0.997 | 1.996 | <0.001 | 1.191 | 0.252 | 1.191 | <0.001 | 1.759 | 0.815 | 1.759 | <0.001 | 0.596 | -0.745 | -1.676 | <0.001 | 0.881 | -0.182 | -1.135 | <0.001 |
| Adaptor-Related Protein Complex 2. Alpha 1 Subunit | **AP2A1** | 1.627 | 0.702 | 1.627 | <0.001 | 1.540 | 0.623 | 1.540 | <0.001 | 1.320 | 0.400 | 1.320 | <0.001 | 0.946 | -0.079 | -1.057 | 0.032 | 0.811 | -0.302 | -1.233 | <0.001 |
| Adaptor-Related Protein Complex 2. Mu 1 Subunit | **AP2M1** | 1.454 | 0.540 | 1.454 | <0.001 | 1.427 | 0.513 | 1.427 | <0.001 | 1.687 | 0.754 | 1.687 | <0.001 | 0.982 | -0.027 | -1.019 | 0.243 | 1.160 | 0.214 | 1.160 | <0.001 |
| Amyloid Beta (A4) Precursor Protein-Binding. Family B. Member 1 | **APBB1** | 1.751 | 0.808 | 1.751 | <0.001 | 1.565 | 0.646 | 1.565 | <0.001 | 1.503 | 0.587 | 1.503 | <0.001 | 0.894 | -0.162 | -1.119 | 0.031 | 0.858 | -0.221 | -1.165 | 0.005 |
| Amyloid Beta (A4) Precursor Protein-Binding. Family B. Member 1 Interacting Protein | **APBB1IP** | 1.476 | 0.561 | 1.476 | <0.001 | 1.576 | 0.657 | 1.576 | <0.001 | 1.451 | 0.537 | 1.451 | <0.001 | 1.068 | 0.095 | 1.068 | 0.284 | 0.984 | -0.024 | -1.017 | 0.799 |
| Amyloid Beta (A4) Precursor Protein-Binding. Family B. Member 2 | **APBB2** | 0.798 | -0.325 | -1.253 | <0.001 | 0.915 | -0.128 | -1.092 | 0.081 | 0.764 | -0.389 | -1.309 | <0.001 | 1.147 | 0.197 | 1.147 | 0.010 | 0.957 | -0.064 | -1.045 | 0.443 |
| Aryl Hydrocarbon Receptor Nuclear Translocator | **ARNT** | 0.705 | -0.504 | -1.418 | <0.001 | 0.577 | -0.795 | -1.735 | <0.001 | 0.619 | -0.692 | -1.615 | <0.001 | 0.818 | -0.291 | -1.223 | <0.001 | 0.878 | -0.187 | -1.139 | 0.002 |
| Activating Transcription Factor 2 | **ATF2** | 0.700 | -0.515 | -1.429 | <0.001 | 0.737 | -0.440 | -1.356 | <0.001 | 0.735 | -0.445 | -1.361 | <0.001 | 1.054 | 0.075 | 1.054 | 0.512 | 1.050 | 0.070 | 1.050 | 0.556 |
| Ataxia Telangiectasia Mutated | **ATM** | 0.646 | -0.630 | -1.548 | <0.001 | 0.665 | -0.589 | -1.504 | <0.001 | 0.740 | -0.434 | -1.351 | <0.001 | 1.029 | 0.041 | 1.029 | 0.559 | 1.146 | 0.197 | 1.146 | 0.005 |
| Atpase. H+ Transporting. Lysosomal 34kda. V1 Subunit D | **ATP6V1D** | 1.132 | 0.179 | 1.132 | 0.034 | 1.251 | 0.323 | 1.251 | <0.001 | 1.100 | 0.137 | 1.100 | 0.119 | 1.104 | 0.143 | 1.104 | 0.070 | 0.971 | -0.042 | -1.030 | 0.619 |
| Ataxia Telangiectasia And Rad3 Related | **ATR** | 0.660 | -0.600 | -1.516 | <0.001 | 1.082 | 0.113 | 1.082 | 0.169 | 0.741 | -0.432 | -1.349 | <0.001 | 1.639 | 0.713 | 1.639 | <0.001 | 1.123 | 0.168 | 1.123 | 0.103 |
| ATR Interacting Protein | **ATRIP** | 1.787 | 0.837 | 1.787 | <0.001 | 2.823 | 1.497 | 2.823 | <0.001 | 1.143 | 0.193 | 1.143 | 0.463 | 1.580 | 0.660 | 1.580 | <0.001 | 0.640 | -0.644 | -1.563 | 0.004 |
| Basic Helix-Loop-Helix Family. Member E40 | **BHLHE40** | 1.645 | 0.718 | 1.645 | <0.001 | 1.254 | 0.327 | 1.254 | <0.001 | 1.088 | 0.121 | 1.088 | 0.109 | 0.762 | -0.391 | -1.312 | <0.001 | 0.661 | -0.597 | -1.512 | <0.001 |
| Basic Helix-Loop-Helix Family. Member E41 | **BHLHE41** | 1.947 | 0.961 | 1.947 | <0.001 | 1.731 | 0.792 | 1.731 | <0.001 | 2.322 | 1.216 | 2.322 | <0.001 | 0.889 | -0.169 | -1.124 | 0.307 | 1.193 | 0.255 | 1.193 | 0.110 |
| Bone Morphogenetic Protein Receptor. Type Ia | **BMPR1A** | 0.643 | -0.638 | -1.556 | <0.001 | 0.780 | -0.358 | -1.281 | <0.001 | 0.658 | -0.604 | -1.520 | <0.001 | 1.214 | 0.280 | 1.214 | <0.001 | 1.024 | 0.034 | 1.024 | 0.634 |
| BCL2/Adenovirus E1B 19kda Interacting Protein 3 | **BNIP3** | 1.381 | 0.465 | 1.381 | <0.001 | 0.875 | -0.192 | -1.143 | <0.001 | 1.049 | 0.069 | 1.049 | 0.181 | 0.634 | -0.658 | -1.578 | <0.001 | 0.760 | -0.396 | -1.316 | <0.001 |
| Beta-Transducin Repeat Containing | **BTRC** | 0.768 | -0.381 | -1.302 | <0.001 | 0.758 | -0.400 | -1.319 | <0.001 | 0.741 | -0.432 | -1.349 | <0.001 | 0.987 | -0.019 | -1.013 | 0.837 | 0.965 | -0.051 | -1.036 | 0.601 |
| Cyclin A2 | **CCNA2** | 1.401 | 0.486 | 1.401 | <0.001 | 2.238 | 1.162 | 2.238 | <0.001 | 0.677 | -0.562 | -1.477 | <0.001 | 1.598 | 0.676 | 1.598 | <0.001 | 0.484 | -1.048 | -2.068 | <0.001 |
| Cyclin B1 | **CCNB1** | 2.007 | 1.005 | 2.007 | <0.001 | 3.064 | 1.616 | 3.064 | <0.001 | 0.520 | -0.944 | -1.925 | <0.001 | 1.527 | 0.611 | 1.527 | <0.001 | 0.259 | -1.949 | -3.862 | <0.001 |
| Cyclin D1 | **CCND1** | 3.936 | 1.977 | 3.936 | <0.001 | 6.376 | 2.673 | 6.376 | <0.001 | 3.530 | 1.820 | 3.530 | <0.001 | 1.620 | 0.696 | 1.620 | <0.001 | 0.897 | -0.157 | -1.115 | <0.001 |
| CCR4 Carbon Catabolite Repression 4-Like | **CCRN4L** | 1.458 | 0.544 | 1.458 | 0.014 | 1.362 | 0.445 | 1.362 | 0.049 | 1.026 | 0.037 | 1.026 | 0.884 | 0.934 | -0.099 | -1.071 | 0.628 | 0.704 | -0.507 | -1.421 | 0.025 |
| Cell Division Cycle 7 Homolog (S. Cerevisiae) | **CDC7** | 0.983 | -0.025 | -1.017 | 0.923 | 1.718 | 0.781 | 1.718 | 0.001 | 0.428 | -1.224 | -2.336 | <0.001 | 1.748 | 0.806 | 1.748 | <0.001 | 0.436 | -1.199 | -2.296 | <0.001 |
| Cyclin-Dependent Kinase 1 | **CDK1** | 1.251 | 0.323 | 1.251 | 0.002 | 2.546 | 1.348 | 2.546 | <0.001 | 0.411 | -1.283 | -2.433 | <0.001 | 2.035 | 1.025 | 2.035 | <0.001 | 0.329 | -1.606 | -3.043 | <0.001 |
| Cyclin-Dependent Kinase 2 Associated Protein 1 | **CDK2AP1** | 1.217 | 0.284 | 1.217 | <0.001 | 1.395 | 0.480 | 1.395 | <0.001 | 1.432 | 0.518 | 1.432 | <0.001 | 1.146 | 0.196 | 1.146 | <0.001 | 1.176 | 0.234 | 1.176 | <0.001 |
| Cyclin-Dependent Kinase Inhibitor 1A (P21. Cip1) | **CDKN1A** | 3.306 | 1.725 | 3.306 | <0.001 | 1.782 | 0.833 | 1.782 | <0.001 | 2.907 | 1.539 | 2.907 | <0.001 | 0.539 | -0.892 | -1.856 | <0.001 | 0.879 | -0.186 | -1.138 | <0.001 |
| CCAAT/Enhancer Binding Protein (C/EBP). Beta | **CEBPB** | 1.215 | 0.281 | 1.215 | <0.001 | 0.899 | -0.153 | -1.112 | 0.054 | 0.758 | -0.400 | -1.320 | <0.001 | 0.740 | -0.434 | -1.351 | <0.001 | 0.623 | -0.682 | -1.604 | <0.001 |
| Carboxylesterase 1 | **CES1** | 0.144 | -2.792 | -6.927 | <0.001 | 0.058 | -4.112 | -17.295 | <0.001 | 0.066 | -3.921 | -15.147 | <0.001 | 0.401 | -1.320 | -2.497 | 0.104 | 0.457 | -1.129 | -2.187 | 0.166 |
| Carboxylesterase 2 | **CES2** | 1.400 | 0.485 | 1.400 | <0.001 | 1.073 | 0.101 | 1.073 | 0.258 | 1.490 | 0.575 | 1.490 | <0.001 | 0.766 | -0.384 | -1.305 | <0.001 | 1.065 | 0.090 | 1.065 | 0.246 |
| CHK2 Checkpoint Homolog (S. Pombe) | **CHEK2** | 0.860 | -0.218 | -1.163 | 0.534 | 1.507 | 0.591 | 1.507 | 0.052 | 1.044 | 0.063 | 1.044 | 0.856 | 1.752 | 0.809 | 1.752 | 0.010 | 1.215 | 0.281 | 1.215 | 0.429 |
| Chromatin Modifying Protein 1A | **CHMP1A** | 1.332 | 0.414 | 1.332 | <0.001 | 1.383 | 0.468 | 1.383 | <0.001 | 1.302 | 0.381 | 1.302 | <0.001 | 1.038 | 0.054 | 1.038 | 0.319 | 0.978 | -0.033 | -1.023 | 0.568 |
| Chromatin Modifying Protein 1B | **CHMP1B** | 0.946 | -0.080 | -1.057 | 0.292 | 0.959 | -0.060 | -1.042 | 0.427 | 0.693 | -0.528 | -1.442 | <0.001 | 1.014 | 0.020 | 1.014 | 0.793 | 0.733 | -0.448 | -1.364 | <0.001 |
| CREB Binding Protein | **CREBBP** | 1.222 | 0.289 | 1.222 | <0.001 | 1.161 | 0.215 | 1.161 | <0.001 | 1.133 | 0.180 | 1.133 | <0.001 | 0.950 | -0.074 | -1.053 | 0.074 | 0.927 | -0.109 | -1.079 | 0.012 |
| Catenin (Cadherin-Associated Protein). Beta 1. 88kda | **CTNNB1** | 0.845 | -0.242 | -1.183 | <0.001 | 0.842 | -0.248 | -1.188 | <0.001 | 0.811 | -0.301 | -1.232 | <0.001 | 0.996 | -0.006 | -1.004 | 0.853 | 0.960 | -0.059 | -1.042 | 0.078 |
| Chemokine (C-X-C Motif) Ligand 12 | **CXCL12** | 0.842 | -0.249 | -1.188 | <0.001 | 0.886 | -0.175 | -1.129 | <0.001 | 0.661 | -0.598 | -1.514 | <0.001 | 1.052 | 0.074 | 1.052 | <0.001 | 0.785 | -0.349 | -1.274 | <0.001 |
| Cytochrome P450. Family 7. Subfamily B. Polypeptide 1 | **CYP7B1** | 0.038 | -4.730 | -26.536 | <0.001 | 0.063 | -3.991 | -15.901 | <0.001 | 0.144 | -2.800 | -6.963 | <0.001 | 1.669 | 0.739 | 1.669 | 0.476 | 3.811 | 1.930 | 3.811 | 0.026 |
| D Site Of Albumin Promoter (Albumin D-Box) Binding Protein | **DBP** | 2.158 | 1.109 | 2.158 | 0.017 | 2.906 | 1.539 | 2.906 | <0.001 | 2.382 | 1.252 | 2.382 | 0.007 | 1.347 | 0.429 | 1.347 | 0.222 | 1.104 | 0.143 | 1.104 | 0.709 |
| DEAD/H (Asp-Glu-Ala-Asp/His) Box Polypeptide 11 | **DDX11** | 1.192 | 0.254 | 1.192 | 0.113 | 2.216 | 1.148 | 2.216 | <0.001 | 0.947 | -0.079 | -1.056 | 0.653 | 1.858 | 0.894 | 1.858 | <0.001 | 0.794 | -0.333 | -1.259 | 0.046 |
| E2F Transcription Factor 1 | **E2F1** | 2.464 | 1.301 | 2.464 | <0.001 | 5.464 | 2.450 | 5.464 | <0.001 | 1.304 | 0.383 | 1.304 | 0.057 | 2.218 | 1.149 | 2.218 | <0.001 | 0.529 | -0.918 | -1.890 | <0.001 |
| Epidermal Growth Factor Receptor | **EGFR** | 0.549 | -0.866 | -1.823 | <0.001 | 0.700 | -0.514 | -1.428 | <0.001 | 0.423 | -1.242 | -2.365 | <0.001 | 1.276 | 0.352 | 1.276 | <0.001 | 0.771 | -0.376 | -1.298 | <0.001 |
| Early Growth Response 1 | **EGR1** | 0.452 | -1.145 | -2.212 | <0.001 | 0.293 | -1.773 | -3.418 | <0.001 | 0.175 | -2.515 | -5.715 | <0.001 | 0.647 | -0.628 | -1.546 | <0.001 | 0.387 | -1.370 | -2.584 | <0.001 |
| Eukaryotic Translation Initiation Factor 2B. Subunit 2 Beta. 39kda | **EIF2B2** | 0.857 | -0.223 | -1.167 | 0.097 | 0.965 | -0.052 | -1.037 | 0.690 | 0.767 | -0.382 | -1.303 | 0.008 | 1.126 | 0.171 | 1.126 | 0.203 | 0.896 | -0.159 | -1.116 | 0.281 |
| Eukaryotic Translation Initiation Factor 2B. Subunit 3 Gamma. 58kda | **EIF2B3** | 1.314 | 0.394 | 1.314 | 0.023 | 1.848 | 0.886 | 1.848 | <0.001 | 1.069 | 0.096 | 1.069 | 0.609 | 1.407 | 0.492 | 1.407 | 0.001 | 0.814 | -0.298 | -1.229 | 0.088 |
| Eukaryotic Translation Initiation Factor 2B. Subunit 4 Delta. 67kda | **EIF2B4** | 1.276 | 0.352 | 1.276 | 0.028 | 1.374 | 0.458 | 1.374 | 0.004 | 1.474 | 0.560 | 1.474 | <0.001 | 1.077 | 0.107 | 1.077 | 0.468 | 1.156 | 0.209 | 1.156 | 0.162 |
| Eukaryotic Translation Initiation Factor 2B. Subunit 5 Epsilon. 82kda | **EIF2B5** | 1.038 | 0.054 | 1.038 | 0.639 | 1.137 | 0.185 | 1.137 | 0.102 | 0.766 | -0.385 | -1.306 | 0.003 | 1.095 | 0.131 | 1.095 | 0.240 | 0.737 | -0.440 | -1.356 | 0.001 |
| ELAV (Embryonic Lethal. Abnormal Vision. Drosophila)-Like 1 (Hu Antigen R) | **ELAVL1** | 1.151 | 0.203 | 1.151 | 0.001 | 1.505 | 0.590 | 1.505 | <0.001 | 0.996 | -0.006 | -1.004 | 0.934 | 1.308 | 0.387 | 1.308 | <0.001 | 0.865 | -0.209 | -1.156 | 0.001 |
| Ecto-NOX Disulfide-Thiol Exchanger 2 | **ENOX2** | 0.676 | -0.565 | -1.479 | <0.001 | 0.903 | -0.148 | -1.108 | 0.307 | 0.746 | -0.422 | -1.340 | 0.007 | 1.335 | 0.417 | 1.335 | 0.008 | 1.104 | 0.142 | 1.104 | 0.407 |
| E1A Binding Protein P300 | **EP300** | 1.105 | 0.144 | 1.105 | <0.001 | 1.079 | 0.110 | 1.079 | 0.008 | 1.112 | 0.153 | 1.112 | <0.001 | 0.977 | -0.034 | -1.024 | 0.393 | 1.007 | 0.009 | 1.007 | 0.818 |
| Epidermal Growth Factor Receptor Pathway Substrate 15 | **EPS15** | 0.643 | -0.637 | -1.555 | <0.001 | 0.626 | -0.675 | -1.596 | <0.001 | 0.615 | -0.701 | -1.625 | <0.001 | 0.974 | -0.038 | -1.027 | 0.567 | 0.957 | -0.064 | -1.045 | 0.356 |
| Estrogen-Related Receptor Alpha | **ESRRA** | 1.297 | 0.375 | 1.297 | 0.003 | 1.507 | 0.592 | 1.507 | <0.001 | 1.111 | 0.151 | 1.111 | 0.258 | 1.162 | 0.217 | 1.162 | 0.052 | 0.857 | -0.223 | -1.167 | 0.073 |
| Fatty Acid Synthase | **FASN** | 1.755 | 0.811 | 1.755 | <0.001 | 2.029 | 1.021 | 2.029 | <0.001 | 1.307 | 0.387 | 1.307 | <0.001 | 1.157 | 0.210 | 1.157 | <0.001 | 0.745 | -0.425 | -1.342 | <0.001 |
| F-Box And Leucine-Rich Repeat Protein 3 | **FBXL3** | 0.550 | -0.862 | -1.817 | <0.001 | 0.640 | -0.644 | -1.562 | <0.001 | 0.503 | -0.991 | -1.987 | <0.001 | 1.163 | 0.218 | 1.163 | 0.001 | 0.914 | -0.129 | -1.094 | 0.076 |
| Farnesyl-Diphosphate Farnesyltransferase 1 | **FDFT1** | 0.783 | -0.353 | -1.277 | <0.001 | 0.834 | -0.262 | -1.199 | <0.001 | 0.769 | -0.379 | -1.300 | <0.001 | 1.065 | 0.091 | 1.065 | 0.147 | 0.982 | -0.026 | -1.018 | 0.698 |
| Fragile X Mental Retardation 1 | **FMR1** | 0.554 | -0.851 | -1.804 | <0.001 | 0.571 | -0.809 | -1.752 | <0.001 | 0.465 | -1.105 | -2.150 | <0.001 | 1.030 | 0.042 | 1.030 | 0.655 | 0.839 | -0.254 | -1.192 | 0.014 |
| Fibronectin 1 | **FN1** | 2.764 | 1.467 | 2.764 | <0.001 | 2.622 | 1.391 | 2.622 | <0.001 | 2.924 | 1.548 | 2.924 | <0.001 | 0.949 | -0.076 | -1.054 | <0.001 | 1.058 | 0.081 | 1.058 | <0.001 |
| FBJ Murine Osteosarcoma Viral Oncogene Homolog | **FOS** | 0.370 | -1.435 | -2.704 | <0.001 | 0.219 | -2.192 | -4.569 | <0.001 | 0.128 | -2.963 | -7.799 | <0.001 | 0.592 | -0.757 | -1.690 | <0.001 | 0.347 | -1.528 | -2.884 | <0.001 |
| Forkhead Box L2 | **FOXL2** | 1.640 | 0.713 | 1.640 | <0.001 | 1.547 | 0.630 | 1.547 | 0.002 | 1.322 | 0.402 | 1.322 | 0.062 | 0.944 | -0.084 | -1.060 | 0.634 | 0.806 | -0.311 | -1.241 | 0.100 |
| Forkhead Box O1 | **FOXO1** | 0.376 | -1.411 | -2.659 | <0.001 | 0.259 | -1.951 | -3.866 | <0.001 | 0.496 | -1.011 | -2.015 | <0.001 | 0.688 | -0.540 | -1.454 | 0.001 | 1.319 | 0.400 | 1.319 | 0.006 |
| GABA(A) Receptor-Associated Protein Like 1 | **GABARAPL1** | 0.985 | -0.022 | -1.015 | 0.582 | 0.466 | -1.101 | -2.144 | <0.001 | 1.179 | 0.238 | 1.179 | <0.001 | 0.474 | -1.078 | -2.112 | <0.001 | 1.198 | 0.260 | 1.198 | <0.001 |
| Growth Arrest And DNA-Damage-Inducible. Alpha | **GADD45A** | 1.358 | 0.442 | 1.358 | <0.001 | 1.531 | 0.615 | 1.531 | <0.001 | 1.289 | 0.366 | 1.289 | 0.001 | 1.128 | 0.173 | 1.128 | 0.056 | 0.949 | -0.076 | -1.054 | 0.439 |
| Glutamate-Cysteine Ligase. Catalytic Subunit | **GCLC** | 1.144 | 0.194 | 1.144 | 0.083 | 1.246 | 0.317 | 1.246 | 0.004 | 1.109 | 0.150 | 1.109 | 0.198 | 1.090 | 0.124 | 1.090 | 0.238 | 0.970 | -0.044 | -1.031 | 0.695 |
| Glyoxalase I | **GLO1** | 0.647 | -0.629 | -1.547 | <0.001 | 0.818 | -0.291 | -1.223 | <0.001 | 0.586 | -0.771 | -1.707 | <0.001 | 1.265 | 0.339 | 1.265 | <0.001 | 0.906 | -0.142 | -1.103 | 0.011 |
| Glutamate-Ammonia Ligase | **GLUL** | 0.520 | -0.943 | -1.923 | <0.001 | 0.656 | -0.608 | -1.524 | <0.001 | 0.512 | -0.966 | -1.953 | <0.001 | 1.262 | 0.335 | 1.262 | <0.001 | 0.985 | -0.022 | -1.016 | 0.671 |
| Guanine Nucleotide Binding Protein (G Protein). Beta Polypeptide 2-Like 1 | **GNB2L1** | 1.169 | 0.226 | 1.169 | <0.001 | 1.049 | 0.069 | 1.049 | 0.006 | 0.999 | -0.001 | -1.001 | 0.963 | 0.897 | -0.157 | -1.115 | <0.001 | 0.854 | -0.227 | -1.170 | <0.001 |
| Glycogen Synthase Kinase 3 Beta | **GSK3B** | 0.858 | -0.220 | -1.165 | <0.001 | 1.042 | 0.059 | 1.042 | 0.193 | 0.943 | -0.085 | -1.060 | 0.079 | 1.213 | 0.279 | 1.213 | <0.001 | 1.099 | 0.136 | 1.099 | 0.006 |
| Glutathione S-Transferase Mu 1 | **GSTM1** | 1.408 | 0.494 | 1.408 | <0.001 | 1.148 | 0.199 | 1.148 | 0.057 | 2.297 | 1.200 | 2.297 | <0.001 | 0.815 | -0.295 | -1.227 | 0.002 | 1.631 | 0.706 | 1.631 | <0.001 |
| Glutathione S-Transferase Pi 1 | **GSTP1** | 1.646 | 0.719 | 1.646 | <0.001 | 1.381 | 0.466 | 1.381 | <0.001 | 1.359 | 0.442 | 1.359 | <0.001 | 0.839 | -0.253 | -1.191 | <0.001 | 0.826 | -0.276 | -1.211 | <0.001 |
| Glutathione S-Transferase Theta 2 | **GSTT2** | 0.962 | -0.056 | -1.040 | 0.710 | 1.068 | 0.095 | 1.068 | 0.517 | 0.980 | -0.029 | -1.020 | 0.853 | 1.110 | 0.151 | 1.110 | 0.304 | 1.019 | 0.027 | 1.019 | 0.861 |
| Glycogen [Starch] Synthase. Muscle | **GYS1** | 1.689 | 0.756 | 1.689 | <0.001 | 1.337 | 0.419 | 1.337 | <0.001 | 1.686 | 0.754 | 1.686 | <0.001 | 0.791 | -0.338 | -1.264 | <0.001 | 0.998 | -0.002 | -1.002 | 0.951 |
| Histone Deacetylase 3 | **HDAC3** | 0.853 | -0.229 | -1.172 | 0.008 | 0.896 | -0.159 | -1.116 | 0.065 | 0.807 | -0.309 | -1.239 | 0.001 | 1.050 | 0.070 | 1.050 | 0.429 | 0.946 | -0.080 | -1.057 | 0.397 |
| Heme Binding Protein 1 | **HEBP1** | 1.024 | 0.035 | 1.024 | 0.694 | 0.816 | -0.294 | -1.226 | 0.002 | 0.921 | -0.119 | -1.086 | 0.202 | 0.796 | -0.328 | -1.256 | <0.001 | 0.899 | -0.154 | -1.112 | 0.095 |
| Hairy And Enhancer Of Split 1. (Drosophila) | **HES1** | 0.497 | -1.008 | -2.011 | <0.001 | 0.580 | -0.786 | -1.724 | <0.001 | 0.218 | -2.195 | -4.579 | <0.001 | 1.166 | 0.222 | 1.166 | 0.148 | 0.439 | -1.187 | -2.277 | <0.001 |
| Hypoxia Inducible Factor 1. Alpha Subunit | **HIF1A** | 0.602 | -0.731 | -1.660 | <0.001 | 0.566 | -0.820 | -1.765 | <0.001 | 0.633 | -0.661 | -1.581 | <0.001 | 0.940 | -0.089 | -1.064 | <0.001 | 1.050 | 0.070 | 1.050 | <0.001 |
| Hexokinase-1 | **HK1** | 1.133 | 0.180 | 1.133 | <0.001 | 1.306 | 0.385 | 1.306 | <0.001 | 0.977 | -0.033 | -1.023 | 0.492 | 1.153 | 0.205 | 1.153 | <0.001 | 0.863 | -0.213 | -1.159 | <0.001 |
| Hexokinase-2 | **HK2** | 1.029 | 0.042 | 1.029 | 0.524 | 0.867 | -0.206 | -1.154 | 0.003 | 1.215 | 0.281 | 1.215 | <0.001 | 0.842 | -0.248 | -1.188 | <0.001 | 1.180 | 0.239 | 1.180 | <0.001 |
| 3-Hydroxy-3-Methylglutaryl-Coa Reductase | **HMGCR** | 0.470 | -1.091 | -2.130 | <0.001 | 0.730 | -0.455 | -1.370 | <0.001 | 0.623 | -0.682 | -1.604 | <0.001 | 1.554 | 0.636 | 1.554 | <0.001 | 1.328 | 0.409 | 1.328 | <0.001 |
| Heat Shock Transcription Factor 1 | **HSF1** | 1.975 | 0.982 | 1.975 | <0.001 | 2.000 | 1.000 | 2.000 | <0.001 | 1.748 | 0.805 | 1.748 | <0.001 | 1.013 | 0.018 | 1.013 | 0.790 | 0.885 | -0.176 | -1.130 | 0.017 |
| Heat Shock Transcription Factor 2 | **HSF2** | 0.778 | -0.361 | -1.285 | 0.008 | 0.853 | -0.230 | -1.173 | 0.084 | 0.568 | -0.815 | -1.760 | <0.001 | 1.096 | 0.132 | 1.096 | 0.348 | 0.730 | -0.454 | -1.370 | 0.005 |
| Heat Shock Protein 90kda Alpha (Cytosolic). Class A Member 1 | **HSP90AA1** | 1.209 | 0.274 | 1.209 | <0.001 | 1.580 | 0.660 | 1.580 | <0.001 | 0.875 | -0.193 | -1.143 | <0.001 | 1.307 | 0.386 | 1.307 | <0.001 | 0.724 | -0.467 | -1.382 | <0.001 |
| Heat Shock 70kda Protein 1A | **HSPA1A** | 1.804 | 0.852 | 1.804 | <0.001 | 1.514 | 0.599 | 1.514 | <0.001 | 1.807 | 0.854 | 1.807 | <0.001 | 0.839 | -0.253 | -1.192 | <0.001 | 1.002 | 0.002 | 1.002 | 0.914 |
| Heat Shock 70kda Protein 5 (Glucose-Regulated Protein. 78kda) | **HSPA5** | 1.077 | 0.107 | 1.077 | <0.001 | 1.464 | 0.550 | 1.464 | <0.001 | 1.268 | 0.343 | 1.268 | <0.001 | 1.359 | 0.443 | 1.359 | <0.001 | 1.178 | 0.236 | 1.178 | <0.001 |
| Heat Shock 60kda Protein 1 (Chaperonin) | **HSPD1** | 1.181 | 0.240 | 1.181 | <0.001 | 1.466 | 0.552 | 1.466 | <0.001 | 0.831 | -0.266 | -1.203 | <0.001 | 1.242 | 0.313 | 1.242 | <0.001 | 0.704 | -0.506 | -1.420 | <0.001 |
| Intercellular Adhesion Molecule 1 | **ICAM1** | 0.308 | -1.700 | -3.249 | <0.001 | 0.155 | -2.688 | -6.446 | <0.001 | 0.208 | -2.264 | -4.802 | <0.001 | 0.504 | -0.988 | -1.984 | <0.001 | 0.677 | -0.564 | -1.478 | 0.003 |
| Inhibitor Of DNA Binding 2. Dominant Negative Helix-Loop-Helix Protein | **ID2** | 1.544 | 0.627 | 1.544 | <0.001 | 1.044 | 0.062 | 1.044 | 0.176 | 1.615 | 0.691 | 1.615 | <0.001 | 0.676 | -0.565 | -1.479 | <0.001 | 1.046 | 0.065 | 1.046 | 0.086 |
| Insulin-Like Growth Factor Binding Protein 3 | **IGFBP3** | 4.810 | 2.266 | 4.810 | <0.001 | 2.573 | 1.363 | 2.573 | <0.001 | 4.261 | 2.091 | 4.261 | <0.001 | 0.535 | -0.903 | -1.869 | <0.001 | 0.886 | -0.175 | -1.129 | <0.001 |
| Insulin-Like Growth Factor Binding Protein 5 | **IGFBP5** | 0.344 | -1.538 | -2.904 | <0.001 | 0.324 | -1.625 | -3.084 | <0.001 | 1.163 | 0.218 | 1.163 | <0.001 | 0.942 | -0.086 | -1.062 | 0.001 | 3.378 | 1.756 | 3.378 | <0.001 |
| Interleukin 6 (Interferon. Beta 2) | **IL6** | 0.420 | -1.251 | -2.380 | <0.001 | 0.233 | -2.104 | -4.299 | <0.001 | 0.439 | -1.186 | -2.276 | <0.001 | 0.553 | -0.853 | -1.807 | <0.001 | 1.046 | 0.064 | 1.046 | 0.664 |
| Insulin Induced Gene 2 | **INSIG2** | 0.688 | -0.541 | -1.455 | <0.001 | 0.736 | -0.443 | -1.359 | 0.001 | 1.013 | 0.019 | 1.013 | 0.876 | 1.070 | 0.098 | 1.070 | 0.487 | 1.474 | 0.560 | 1.474 | <0.001 |
| Interferon Regulatory Factor 7 | **IRF7** | 1.505 | 0.589 | 1.505 | 0.031 | 0.947 | -0.079 | -1.056 | 0.797 | 1.008 | 0.011 | 1.008 | 0.972 | 0.629 | -0.668 | -1.589 | 0.015 | 0.670 | -0.579 | -1.493 | 0.039 |
| Integrin Alpha FG-GAP Repeat Containing 2 | **ITFG2** | 1.245 | 0.316 | 1.245 | 0.037 | 1.144 | 0.194 | 1.144 | 0.210 | 1.133 | 0.181 | 1.133 | 0.260 | 0.919 | -0.122 | -1.089 | 0.400 | 0.910 | -0.136 | -1.099 | 0.368 |
| Integrin. Alpha 5 (Fibronectin Receptor. Alpha Polypeptide) | **ITGA5** | 1.725 | 0.787 | 1.725 | <0.001 | 2.209 | 1.144 | 2.209 | <0.001 | 1.566 | 0.647 | 1.566 | <0.001 | 1.281 | 0.357 | 1.281 | <0.001 | 0.907 | -0.140 | -1.102 | <0.001 |
| Jun Proto-Oncogene | **JUN** | 1.334 | 0.416 | 1.334 | <0.001 | 1.357 | 0.441 | 1.357 | <0.001 | 0.921 | -0.119 | -1.086 | 0.017 | 1.017 | 0.025 | 1.017 | 0.537 | 0.690 | -0.535 | -1.449 | <0.001 |
| Kelch-Like ECH-Associated Protein 1 | **KEAP1** | 1.652 | 0.724 | 1.652 | <0.001 | 1.428 | 0.514 | 1.428 | <0.001 | 1.468 | 0.554 | 1.468 | <0.001 | 0.865 | -0.210 | -1.157 | 0.003 | 0.889 | -0.170 | -1.125 | 0.021 |
| KIT Ligand | **KITLG** | 0.770 | -0.377 | -1.298 | <0.001 | 1.051 | 0.072 | 1.051 | 0.039 | 0.797 | -0.327 | -1.254 | <0.001 | 1.365 | 0.449 | 1.365 | <0.001 | 1.035 | 0.050 | 1.035 | 0.222 |
| Kruppel-Like Factor 10 | **KLF10** | 0.809 | -0.305 | -1.236 | <0.001 | 0.999 | -0.002 | -1.001 | 0.971 | 0.849 | -0.236 | -1.177 | <0.001 | 1.234 | 0.303 | 1.234 | <0.001 | 1.049 | 0.070 | 1.049 | 0.291 |
| Lactate Dehydrogenase A | **LDHA** | 1.131 | 0.177 | 1.131 | <0.001 | 1.055 | 0.078 | 1.055 | <0.001 | 1.008 | 0.012 | 1.008 | 0.368 | 0.933 | -0.100 | -1.072 | <0.001 | 0.891 | -0.166 | -1.122 | <0.001 |
| Low Density Lipoprotein Receptor | **LDLR** | 0.631 | -0.665 | -1.586 | <0.001 | 0.753 | -0.410 | -1.329 | <0.001 | 0.471 | -1.087 | -2.124 | <0.001 | 1.193 | 0.255 | 1.193 | <0.001 | 0.747 | -0.422 | -1.339 | <0.001 |
| Lectin. Galactoside-Binding. Soluble. 9 | **LGALS9** | 0.450 | -1.152 | -2.223 | <0.001 | 0.203 | -2.303 | -4.933 | <0.001 | 0.566 | -0.822 | -1.767 | 0.002 | 0.451 | -1.150 | -2.219 | 0.003 | 1.258 | 0.331 | 1.258 | 0.294 |
| Lectin. Mannose-Binding. 1 | **LMAN1** | 0.514 | -0.961 | -1.947 | <0.001 | 0.618 | -0.695 | -1.619 | <0.001 | 0.655 | -0.610 | -1.527 | <0.001 | 1.202 | 0.266 | 1.202 | <0.001 | 1.275 | 0.351 | 1.275 | <0.001 |
| Lectin. Mannose-Binding 2 | **LMAN2** | 1.846 | 0.884 | 1.846 | <0.001 | 1.620 | 0.696 | 1.620 | <0.001 | 1.527 | 0.611 | 1.527 | <0.001 | 0.878 | -0.188 | -1.139 | <0.001 | 0.827 | -0.273 | -1.209 | <0.001 |
| Lipin 1 | **LPIN1** | 0.683 | -0.549 | -1.463 | <0.001 | 0.735 | -0.445 | -1.361 | <0.001 | 0.722 | -0.471 | -1.386 | <0.001 | 1.075 | 0.104 | 1.075 | 0.180 | 1.056 | 0.078 | 1.056 | 0.331 |
| MAP-Kinase Activating Death Domain | **MADD** | 1.125 | 0.170 | 1.125 | 0.029 | 0.999 | -0.002 | -1.001 | 0.984 | 1.116 | 0.159 | 1.116 | 0.049 | 0.888 | -0.172 | -1.126 | 0.026 | 0.992 | -0.011 | -1.008 | 0.883 |
| Monoamine Oxidase A | **MAOA** | 0.192 | -2.380 | -5.205 | <0.001 | 0.060 | -4.066 | -16.750 | <0.001 | 0.333 | -1.585 | -3.001 | <0.001 | 0.311 | -1.686 | -3.218 | 0.001 | 1.735 | 0.795 | 1.735 | 0.020 |
| Mitogen-Activated Protein Kinase 3 | **MAPK3** | 1.055 | 0.077 | 1.055 | 0.200 | 0.856 | -0.224 | -1.168 | <0.001 | 1.023 | 0.032 | 1.023 | 0.605 | 0.812 | -0.301 | -1.232 | <0.001 | 0.970 | -0.045 | -1.031 | 0.467 |
| Mannan-Binding Lectin Serine Peptidase 1 (C4/C2 Activating Component Of Ra-Reactive Factor) | **MASP1** | 0.187 | -2.416 | -5.336 | <0.001 | 0.088 | -3.508 | -11.375 | <0.001 | 0.132 | -2.925 | -7.593 | <0.001 | 0.469 | -1.092 | -2.132 | <0.001 | 0.703 | -0.509 | -1.423 | <0.001 |
| Mdm2 P53 Binding Protein Homolog (Mouse) | **MDM2** | 1.229 | 0.297 | 1.229 | <0.001 | 1.207 | 0.271 | 1.207 | <0.001 | 1.335 | 0.417 | 1.335 | <0.001 | 0.982 | -0.026 | -1.019 | 0.601 | 1.086 | 0.119 | 1.086 | 0.019 |
| Microsomal Glutathione S-Transferase 1 | **MGST1** | 0.595 | -0.749 | -1.681 | <0.001 | 0.669 | -0.579 | -1.494 | <0.001 | 0.593 | -0.754 | -1.687 | <0.001 | 1.125 | 0.170 | 1.125 | 0.003 | 0.996 | -0.005 | -1.004 | 0.930 |
| V-Myc Myelocytomatosis Viral Oncogene Homolog (Avian) | **MYC** | 0.903 | -0.147 | -1.108 | 0.036 | 1.010 | 0.014 | 1.010 | 0.840 | 0.659 | -0.601 | -1.516 | <0.001 | 1.118 | 0.161 | 1.118 | 0.021 | 0.730 | -0.453 | -1.369 | <0.001 |
| Nascent Polypeptide-Associated Complex Alpha Subunit | **NACA** | 0.936 | -0.095 | -1.068 | 0.023 | 0.941 | -0.087 | -1.062 | 0.038 | 0.863 | -0.213 | -1.159 | <0.001 | 1.006 | 0.008 | 1.006 | 0.848 | 0.922 | -0.117 | -1.085 | 0.009 |
| Nicotinamide Phosphoribosyltransferase | **NAMPT** | 0.301 | -1.731 | -3.320 | <0.001 | 0.309 | -1.695 | -3.237 | <0.001 | 0.281 | -1.829 | -3.553 | <0.001 | 1.025 | 0.036 | 1.025 | 0.690 | 0.934 | -0.098 | -1.070 | 0.309 |
| Nuclear Receptor Co-Repressor 1 | **NCOR1** | 0.868 | -0.204 | -1.152 | <0.001 | 0.910 | -0.136 | -1.099 | 0.001 | 0.876 | -0.192 | -1.142 | <0.001 | 1.048 | 0.068 | 1.048 | 0.101 | 1.008 | 0.012 | 1.008 | 0.778 |
| Nuclear Factor (Erythroid-Derived 2)-Like 2 | **NFE2L2** | 0.672 | -0.573 | -1.487 | <0.001 | 0.586 | -0.770 | -1.705 | <0.001 | 0.702 | -0.509 | -1.424 | <0.001 | 0.872 | -0.197 | -1.147 | 0.001 | 1.045 | 0.063 | 1.045 | 0.265 |
| Nuclear Factor. Interleukin 3 Regulated | **NFIL3** | 0.423 | -1.241 | -2.364 | <0.001 | 0.409 | -1.289 | -2.444 | <0.001 | 0.355 | -1.494 | -2.818 | <0.001 | 0.967 | -0.048 | -1.034 | 0.661 | 0.839 | -0.253 | -1.192 | 0.031 |
| Nuclear Factor Of Kappa Light Polypeptide Gene Enhancer In B-Cells Inhibitor. Alpha | **NFKBIA** | 0.593 | -0.755 | -1.688 | <0.001 | 0.484 | -1.046 | -2.065 | <0.001 | 0.811 | -0.302 | -1.233 | 0.009 | 0.817 | -0.291 | -1.224 | 0.043 | 1.369 | 0.453 | 1.369 | 0.001 |
| Nuclear Transcription Factor Y. Alpha | **NFYA** | 0.977 | -0.034 | -1.024 | 0.702 | 1.231 | 0.300 | 1.231 | <0.001 | 0.969 | -0.046 | -1.032 | 0.624 | 1.260 | 0.334 | 1.260 | <0.001 | 0.992 | -0.011 | -1.008 | 0.903 |
| Nuclear Transcription Factor Y. Beta | **NFYB** | 0.379 | -1.400 | -2.639 | <0.001 | 0.255 | -1.970 | -3.918 | <0.001 | 0.355 | -1.493 | -2.816 | <0.001 | 0.674 | -0.570 | -1.485 | <0.001 | 0.937 | -0.093 | -1.067 | 0.427 |
| Nicotinamide N-Methyltransferase | **NNMT** | 0.745 | -0.425 | -1.343 | <0.001 | 0.417 | -1.263 | -2.400 | <0.001 | 0.671 | -0.576 | -1.491 | <0.001 | 0.560 | -0.838 | -1.787 | <0.001 | 0.901 | -0.151 | -1.110 | <0.001 |
| Non-POU Domain Containing. Octamer-Binding | **NONO** | 0.935 | -0.096 | -1.069 | 0.003 | 1.092 | 0.127 | 1.092 | <0.001 | 0.868 | -0.204 | -1.152 | <0.001 | 1.167 | 0.223 | 1.167 | <0.001 | 0.928 | -0.108 | -1.078 | 0.002 |
| Notch Homolog 1. Translocation-Associated (Drosophila) | **NOTCH1** | 2.769 | 1.469 | 2.769 | <0.001 | 2.605 | 1.382 | 2.605 | <0.001 | 1.227 | 0.295 | 1.227 | 0.012 | 0.941 | -0.088 | -1.063 | 0.227 | 0.443 | -1.175 | -2.257 | <0.001 |
| Niemann-Pick Disease. Type C1 | **NPC1** | 0.780 | -0.359 | -1.283 | <0.001 | 0.913 | -0.131 | -1.095 | 0.002 | 0.585 | -0.775 | -1.711 | <0.001 | 1.171 | 0.228 | 1.171 | <0.001 | 0.750 | -0.416 | -1.334 | <0.001 |
| Nuclear Receptor Subfamily 2. Group F. Member 6 | **NR2F6** | 1.263 | 0.337 | 1.263 | 0.005 | 1.325 | 0.406 | 1.325 | 0.001 | 1.294 | 0.372 | 1.294 | 0.002 | 1.049 | 0.069 | 1.049 | 0.531 | 1.025 | 0.035 | 1.025 | 0.759 |
| Nuclear Respiratory Factor 1 | **NRF1** | 1.465 | 0.551 | 1.465 | 0.002 | 1.439 | 0.525 | 1.439 | 0.004 | 0.998 | -0.002 | -1.002 | 0.992 | 0.982 | -0.026 | -1.018 | 0.874 | 0.682 | -0.553 | -1.467 | 0.003 |
| Prolyl 4-Hydroxylase. Alpha Polypeptide I | **P4HA1** | 1.001 | 0.002 | 1.001 | 0.948 | 0.824 | -0.279 | -1.213 | <0.001 | 1.017 | 0.025 | 1.017 | 0.441 | 0.823 | -0.281 | -1.215 | <0.001 | 1.016 | 0.023 | 1.016 | 0.475 |
| Prolyl 4-Hydroxylase. Alpha Polypeptide Ii | **P4HA2** | 1.946 | 0.961 | 1.946 | <0.001 | 1.990 | 0.993 | 1.990 | <0.001 | 1.919 | 0.940 | 1.919 | <0.001 | 1.023 | 0.032 | 1.023 | 0.415 | 0.986 | -0.020 | -1.014 | 0.624 |
| Poly (ADP-Ribose) Polymerase 1 | **PARP1** | 0.710 | -0.494 | -1.409 | <0.001 | 0.923 | -0.115 | -1.083 | 0.083 | 0.581 | -0.785 | -1.723 | <0.001 | 1.301 | 0.379 | 1.301 | <0.001 | 0.818 | -0.290 | -1.223 | 0.001 |
| Pterin-4 Alpha-Carbinolamine Dehydratase/Dimerization Cofactor Of Hepatocyte Nuclear Factor 1 Alpha | **PCBD1** | 1.281 | 0.357 | 1.281 | 0.025 | 1.098 | 0.135 | 1.098 | 0.416 | 1.176 | 0.234 | 1.176 | 0.166 | 0.858 | -0.222 | -1.166 | 0.152 | 0.918 | -0.123 | -1.089 | 0.435 |
| Phosphoenolpyruvate Carboxykinase 2 (Mitochondrial) | **PCK2** | 1.501 | 0.585 | 1.501 | <0.001 | 1.163 | 0.218 | 1.163 | 0.003 | 1.306 | 0.385 | 1.306 | <0.001 | 0.775 | -0.367 | -1.290 | <0.001 | 0.870 | -0.200 | -1.149 | 0.003 |
| Purkinje Cell Protein 4 | **PCP4** | 0.051 | -4.296 | -19.644 | <0.001 | 0.026 | -5.248 | -38.000 | <0.001 | 0.026 | -5.248 | -38.000 | <0.001 | 0.500 | -1.000 | -2.000 | 0.239 | 0.500 | -1.000 | -2.000 | 0.262 |
| Platelet-Derived Growth Factor Receptor. Alpha Polypeptide | **PDGFRA** | 0.441 | -1.182 | -2.269 | <0.001 | 0.352 | -1.508 | -2.844 | <0.001 | 0.418 | -1.258 | -2.391 | <0.001 | 0.798 | -0.326 | -1.254 | <0.001 | 0.949 | -0.076 | -1.054 | 0.017 |
| Platelet-Derived Growth Factor Receptor. Beta Polypeptide | **PDGFRB** | 0.549 | -0.866 | -1.823 | <0.001 | 0.377 | -1.407 | -2.652 | <0.001 | 0.665 | -0.589 | -1.504 | <0.001 | 0.687 | -0.541 | -1.455 | <0.001 | 1.212 | 0.277 | 1.212 | <0.001 |
| Pyruvate Kinase. Muscle | **PKM2** | 1.609 | 0.686 | 1.609 | <0.001 | 1.645 | 0.718 | 1.645 | <0.001 | 1.627 | 0.702 | 1.627 | <0.001 | 1.023 | 0.033 | 1.023 | 0.004 | 1.011 | 0.016 | 1.011 | 0.159 |
| Plasminogen Activator. Tissue | **PLAT** | 0.780 | -0.358 | -1.282 | 0.010 | 1.290 | 0.367 | 1.290 | 0.003 | 0.962 | -0.056 | -1.040 | 0.682 | 1.653 | 0.725 | 1.653 | <0.001 | 1.233 | 0.302 | 1.233 | 0.037 |
| Polymerase (DNA Directed). Alpha 1. Catalytic Subunit | **POLA1** | 0.918 | -0.124 | -1.090 | 0.427 | 1.332 | 0.413 | 1.332 | 0.004 | 0.603 | -0.729 | -1.658 | <0.001 | 1.451 | 0.537 | 1.451 | <0.001 | 0.657 | -0.606 | -1.522 | 0.001 |
| P450 (Cytochrome) Oxidoreductase | **POR** | 1.247 | 0.318 | 1.247 | 0.005 | 1.215 | 0.281 | 1.215 | 0.014 | 1.033 | 0.046 | 1.033 | 0.705 | 0.974 | -0.038 | -1.026 | 0.725 | 0.828 | -0.272 | -1.208 | 0.018 |
| POU Class 2 Homeobox 1 | **POU2F1** | 1.253 | 0.325 | 1.253 | 0.259 | 1.233 | 0.302 | 1.233 | 0.298 | 1.860 | 0.895 | 1.860 | 0.001 | 0.984 | -0.024 | -1.017 | 0.931 | 1.484 | 0.570 | 1.484 | 0.024 |
| Peroxisome Proliferator-Activated Receptor Alpha | **PPARA** | 0.594 | -0.752 | -1.684 | <0.001 | 0.687 | -0.542 | -1.456 | <0.001 | 0.746 | -0.422 | -1.340 | <0.001 | 1.157 | 0.211 | 1.157 | 0.007 | 1.257 | 0.330 | 1.257 | <0.001 |
| Peroxisome Proliferator-Activated Receptor Delta | **PPARD** | 1.162 | 0.216 | 1.162 | 0.001 | 1.403 | 0.489 | 1.403 | <0.001 | 1.202 | 0.265 | 1.202 | <0.001 | 1.208 | 0.273 | 1.208 | <0.001 | 1.034 | 0.049 | 1.034 | 0.458 |
| Peroxisome Proliferator-Activated Receptor Gamma | **PPARG** | 0.242 | -2.048 | -4.136 | <0.001 | 0.320 | -1.646 | -3.129 | <0.001 | 0.232 | -2.106 | -4.306 | <0.001 | 1.322 | 0.402 | 1.322 | 0.290 | 0.960 | -0.058 | -1.041 | 0.891 |
| Protein Phosphatase 5. Catalytic Subunit | **PPP5C** | 1.324 | 0.405 | 1.324 | <0.001 | 1.242 | 0.312 | 1.242 | 0.002 | 0.905 | -0.144 | -1.105 | 0.205 | 0.938 | -0.092 | -1.066 | 0.324 | 0.684 | -0.549 | -1.463 | <0.001 |
| Peroxiredoxin 2 | **PRDX2** | 2.254 | 1.173 | 2.254 | <0.001 | 1.491 | 0.576 | 1.491 | <0.001 | 1.554 | 0.636 | 1.554 | <0.001 | 0.661 | -0.597 | -1.512 | <0.001 | 0.689 | -0.536 | -1.450 | <0.001 |
| Protein Kinase. Amp-Activated. Alpha 1 Catalytic Subunit | **PRKAA1** | 0.649 | -0.623 | -1.540 | <0.001 | 0.680 | -0.556 | -1.470 | <0.001 | 0.680 | -0.557 | -1.471 | <0.001 | 1.047 | 0.067 | 1.047 | 0.271 | 1.046 | 0.066 | 1.046 | 0.295 |
| Protein Kinase C. Alpha | **PRKCA** | 0.858 | -0.221 | -1.166 | <0.001 | 0.916 | -0.127 | -1.092 | 0.002 | 0.899 | -0.154 | -1.112 | <0.001 | 1.067 | 0.094 | 1.067 | 0.029 | 1.048 | 0.068 | 1.048 | 0.131 |
| Prion Protein | **PRNP** | 1.374 | 0.458 | 1.374 | <0.001 | 1.389 | 0.474 | 1.389 | <0.001 | 1.339 | 0.421 | 1.339 | <0.001 | 1.011 | 0.016 | 1.011 | 0.533 | 0.975 | -0.037 | -1.026 | 0.157 |
| Prostaglandin D2 Synthase 21kda (Brain) | **PTGDS** | 0.720 | -0.474 | -1.389 | 0.157 | 0.225 | -2.151 | -4.440 | <0.001 | 0.463 | -1.111 | -2.160 | 0.004 | 0.313 | -1.676 | -3.196 | 0.001 | 0.643 | -0.637 | -1.555 | 0.128 |
| Prostaglandin-Endoperoxide Synthase 2 (Prostaglandin G/H Synthase And Cyclooxygenase) COX2 | **PTGS2** | 2.003 | 1.002 | 2.003 | <0.001 | 3.307 | 1.725 | 3.307 | <0.001 | 1.725 | 0.787 | 1.725 | <0.001 | 1.651 | 0.723 | 1.651 | <0.001 | 0.861 | -0.216 | -1.161 | <0.001 |
| Phosphorylase. Glycogen. Liver | **PYGL** | 0.574 | -0.801 | -1.743 | <0.001 | 0.302 | -1.726 | -3.309 | <0.001 | 0.668 | -0.583 | -1.498 | <0.001 | 0.527 | -0.925 | -1.898 | <0.001 | 1.164 | 0.219 | 1.164 | 0.008 |
| Retinoic Acid Receptor. Alpha | **RARA** | 1.564 | 0.645 | 1.564 | <0.001 | 0.976 | -0.036 | -1.025 | 0.723 | 1.181 | 0.240 | 1.181 | 0.015 | 0.624 | -0.681 | -1.603 | <0.001 | 0.755 | -0.405 | -1.324 | <0.001 |
| Retinoblastoma 1 | **RB1** | 0.528 | -0.922 | -1.895 | <0.001 | 0.736 | -0.443 | -1.359 | <0.001 | 0.548 | -0.868 | -1.826 | <0.001 | 1.394 | 0.479 | 1.394 | <0.001 | 1.038 | 0.053 | 1.038 | 0.482 |
| Ring-Box 1. E3 Ubiquitin Protein Ligase | **RBX1** | 1.076 | 0.106 | 1.076 | 0.088 | 1.136 | 0.185 | 1.136 | 0.002 | 0.879 | -0.187 | -1.138 | 0.006 | 1.056 | 0.079 | 1.056 | 0.184 | 0.817 | -0.292 | -1.225 | <0.001 |
| Regulator Of Chromosome Condensation 1 | **RCC1** | 0.994 | -0.009 | -1.006 | 0.924 | 1.674 | 0.744 | 1.674 | <0.001 | 0.937 | -0.094 | -1.067 | 0.339 | 1.685 | 0.752 | 1.685 | <0.001 | 0.943 | -0.085 | -1.061 | 0.384 |
| Retinoid X Receptor. Alpha | **RXRA** | 1.562 | 0.643 | 1.562 | <0.001 | 1.605 | 0.683 | 1.605 | <0.001 | 1.402 | 0.487 | 1.402 | <0.001 | 1.028 | 0.040 | 1.028 | 0.404 | 0.898 | -0.156 | -1.114 | 0.002 |
| Secretogranin V (7B2 Protein) | **SCG5** | 1.518 | 0.602 | 1.518 | 0.005 | 2.906 | 1.539 | 2.906 | <0.001 | 1.674 | 0.743 | 1.674 | 0.001 | 1.914 | 0.937 | 1.914 | <0.001 | 1.103 | 0.141 | 1.103 | 0.466 |
| Sec61 Alpha 1 Subunit | **SEC61A1** | 1.007 | 0.010 | 1.007 | 0.597 | 1.135 | 0.183 | 1.135 | <0.001 | 1.132 | 0.179 | 1.132 | <0.001 | 1.127 | 0.173 | 1.127 | <0.001 | 1.124 | 0.169 | 1.124 | <0.001 |
| Sec61 Gamma Subunit | **SEC61G** | 1.045 | 0.063 | 1.045 | 0.354 | 1.062 | 0.087 | 1.062 | 0.202 | 0.885 | -0.177 | -1.130 | 0.016 | 1.016 | 0.023 | 1.016 | 0.725 | 0.847 | -0.240 | -1.181 | 0.001 |
| Pai-1 | **SERPINE1** | 5.146 | 2.363 | 5.146 | <0.001 | 8.616 | 3.107 | 8.616 | <0.001 | 3.570 | 1.836 | 3.570 | <0.001 | 1.674 | 0.744 | 1.674 | <0.001 | 0.694 | -0.527 | -1.441 | <0.001 |
| Sphingomyelin Synthase 2 | **SGMS2** | 0.588 | -0.767 | -1.702 | <0.001 | 0.778 | -0.361 | -1.285 | <0.001 | 0.724 | -0.467 | -1.382 | <0.001 | 1.325 | 0.406 | 1.325 | <0.001 | 1.231 | 0.300 | 1.231 | 0.001 |
| S-Phase Kinase-Associated Protein 1 | **SKP1** | 0.697 | -0.521 | -1.435 | <0.001 | 0.918 | -0.124 | -1.090 | 0.001 | 1.056 | 0.079 | 1.056 | 0.034 | 1.317 | 0.397 | 1.317 | <0.001 | 1.515 | 0.600 | 1.515 | <0.001 |
| Solute Carrier Family 22. Member 15 | **SLC22A15** | 0.744 | -0.427 | -1.345 | <0.001 | 0.654 | -0.613 | -1.530 | <0.001 | 0.660 | -0.599 | -1.515 | <0.001 | 0.879 | -0.186 | -1.137 | 0.167 | 0.888 | -0.172 | -1.127 | 0.216 |
| Solute Carrier Family 25 (Mitochondrial Carrier; Citrate Transporter). Member 1 | **SLC25A1** | 1.678 | 0.747 | 1.678 | <0.001 | 1.294 | 0.372 | 1.294 | <0.001 | 1.247 | 0.319 | 1.247 | 0.001 | 0.772 | -0.374 | -1.296 | <0.001 | 0.743 | -0.428 | -1.345 | <0.001 |
| Solute Carrier Family 27 (Fatty Acid Transporter). Member 1 | **SLC27A1** | 1.992 | 0.995 | 1.992 | <0.001 | 1.108 | 0.148 | 1.108 | 0.236 | 1.553 | 0.635 | 1.553 | <0.001 | 0.556 | -0.847 | -1.798 | <0.001 | 0.779 | -0.360 | -1.283 | <0.001 |
| Solute Carrier Family 29 (Nucleoside Transporters). Member 1 | **SLC29A1** | 0.754 | -0.407 | -1.326 | 0.034 | 1.449 | 0.535 | 1.449 | 0.001 | 0.670 | -0.577 | -1.491 | 0.005 | 1.922 | 0.942 | 1.922 | <0.001 | 0.889 | -0.169 | -1.125 | 0.439 |
| Solute Carrier Family 39 (Zinc Transporter). Member 1 | **SLC39A1** | 1.042 | 0.060 | 1.042 | 0.363 | 0.856 | -0.225 | -1.169 | 0.001 | 0.990 | -0.014 | -1.010 | 0.836 | 0.821 | -0.285 | -1.218 | <0.001 | 0.950 | -0.074 | -1.053 | 0.273 |
| Solute Carrier Family 41. Member 3 | **SLC41A3** | 1.260 | 0.334 | 1.260 | 0.001 | 1.111 | 0.152 | 1.111 | 0.147 | 1.259 | 0.333 | 1.259 | 0.002 | 0.882 | -0.182 | -1.134 | 0.064 | 0.999 | -0.001 | -1.001 | 0.990 |
| Solute Carrier Family 7 (Amino Acid Transporter Light Chain. L System). Member 8 | **SLC7A8** | 1.778 | 0.830 | 1.778 | <0.001 | 2.807 | 1.489 | 2.807 | <0.001 | 1.982 | 0.987 | 1.982 | <0.001 | 1.579 | 0.659 | 1.579 | <0.001 | 1.115 | 0.157 | 1.115 | 0.003 |
| Solute Carrier Family 9 (Sodium/Hydrogen Exchanger). Member 3 Regulator 2 | **SLC9A3R2** | 2.191 | 1.132 | 2.191 | <0.001 | 1.186 | 0.246 | 1.186 | 0.076 | 0.959 | -0.060 | -1.043 | 0.691 | 0.541 | -0.886 | -1.848 | <0.001 | 0.438 | -1.192 | -2.284 | <0.001 |
| Solute Carrier Family 9 (Sodium/Hydrogen Exchanger). Member 8 | **SLC9A8** | 1.066 | 0.093 | 1.066 | 0.299 | 1.144 | 0.194 | 1.144 | 0.027 | 1.275 | 0.350 | 1.275 | <0.001 | 1.073 | 0.101 | 1.073 | 0.238 | 1.195 | 0.258 | 1.195 | 0.003 |
| Solute Carrier Family 9 (Sodium/Hydrogen Exchanger). Member 9 | **SLC9A9** | 0.332 | -1.589 | -3.009 | <0.001 | 0.297 | -1.751 | -3.366 | <0.001 | 0.231 | -2.116 | -4.335 | <0.001 | 0.894 | -0.162 | -1.119 | 0.565 | 0.694 | -0.527 | -1.441 | 0.089 |
| SMAD Family Member 4 | **SMAD4** | 0.620 | -0.691 | -1.614 | <0.001 | 0.651 | -0.619 | -1.536 | <0.001 | 0.617 | -0.698 | -1.622 | <0.001 | 1.051 | 0.071 | 1.051 | 0.259 | 0.995 | -0.007 | -1.005 | 0.919 |
| Superoxide Dismutase 1. Soluble | **SOD1** | 1.816 | 0.861 | 1.816 | <0.001 | 1.497 | 0.582 | 1.497 | <0.001 | 1.425 | 0.511 | 1.425 | <0.001 | 0.824 | -0.279 | -1.213 | 0.005 | 0.785 | -0.350 | -1.274 | 0.001 |
| Sp1 Transcription Factor | **SP1** | 0.691 | -0.532 | -1.446 | <0.001 | 0.712 | -0.491 | -1.405 | <0.001 | 0.713 | -0.487 | -1.402 | <0.001 | 1.029 | 0.041 | 1.029 | 0.412 | 1.032 | 0.045 | 1.032 | 0.384 |
| Secreted Phosphoprotein 1 | **SPP1** | 36.029 | 5.171 | 36.029 | <0.001 | 32.203 | 5.009 | 32.203 | <0.001 | 9.953 | 3.315 | 9.953 | <0.001 | 0.894 | -0.162 | -1.119 | 0.346 | 0.276 | -1.856 | -3.620 | <0.001 |
| Sterol Regulatory Element Binding Transcription Factor 1(SREBP-1C) | **SREBF1** | 1.220 | 0.286 | 1.220 | <0.001 | 0.935 | -0.098 | -1.070 | 0.205 | 0.901 | -0.150 | -1.110 | 0.062 | 0.766 | -0.384 | -1.305 | <0.001 | 0.739 | -0.437 | -1.353 | <0.001 |
| Serum Response Factor (C-Fos Serum Response Element-Binding Transcription Factor) | **SRF** | 1.267 | 0.341 | 1.267 | <0.001 | 1.037 | 0.053 | 1.037 | 0.398 | 1.273 | 0.348 | 1.273 | <0.001 | 0.819 | -0.288 | -1.221 | <0.001 | 1.005 | 0.007 | 1.005 | 0.905 |
| Signal Recognition Particle 14kda (Homologous Alu RNA Binding Protein) | **SRP14** | 0.557 | -0.844 | -1.795 | <0.001 | 0.687 | -0.541 | -1.455 | <0.001 | 0.641 | -0.641 | -1.560 | <0.001 | 1.234 | 0.303 | 1.234 | <0.001 | 1.151 | 0.203 | 1.151 | <0.001 |
| Signal Recognition Particle 9kda | **SRP9** | 0.877 | -0.190 | -1.141 | <0.001 | 0.892 | -0.165 | -1.121 | <0.001 | 0.690 | -0.535 | -1.449 | <0.001 | 1.017 | 0.024 | 1.017 | 0.498 | 0.787 | -0.346 | -1.271 | <0.001 |
| SRR1 Domain Containing | **SRRD** | 0.740 | -0.434 | -1.351 | 0.005 | 0.776 | -0.366 | -1.289 | 0.017 | 0.654 | -0.612 | -1.528 | <0.001 | 1.048 | 0.068 | 1.048 | 0.681 | 0.884 | -0.178 | -1.131 | 0.318 |
| Signal Transducer And Activator Of Transcription 1 | **STAT1** | 0.681 | -0.554 | -1.469 | <0.001 | 0.396 | -1.336 | -2.525 | <0.001 | 0.781 | -0.357 | -1.281 | <0.001 | 0.582 | -0.782 | -1.719 | <0.001 | 1.146 | 0.197 | 1.146 | <0.001 |
| Signal Transducer And Activator Of Transcription 5A | **STAT5A** | 0.628 | -0.670 | -1.591 | <0.001 | 0.425 | -1.236 | -2.355 | <0.001 | 0.606 | -0.722 | -1.650 | <0.001 | 0.676 | -0.565 | -1.480 | <0.001 | 0.965 | -0.052 | -1.037 | 0.703 |
| Syntaxin Binding Protein 3 | **STXBP3** | 0.578 | -0.791 | -1.731 | <0.001 | 0.560 | -0.837 | -1.787 | <0.001 | 0.606 | -0.724 | -1.651 | <0.001 | 0.969 | -0.046 | -1.032 | 0.711 | 1.048 | 0.068 | 1.048 | 0.590 |
| SMT3 Suppressor Of Mif Two 3 Homolog 3 (S. Cerevisiae) | **SUMO3** | 1.180 | 0.239 | 1.180 | <0.001 | 1.224 | 0.291 | 1.224 | <0.001 | 0.957 | -0.063 | -1.045 | 0.310 | 1.037 | 0.052 | 1.037 | 0.334 | 0.811 | -0.302 | -1.233 | <0.001 |
| TATA Binding Protein | **TBP** | 1.567 | 0.648 | 1.567 | <0.001 | 1.381 | 0.466 | 1.381 | 0.010 | 1.208 | 0.273 | 1.208 | 0.155 | 0.881 | -0.183 | -1.135 | 0.252 | 0.771 | -0.375 | -1.297 | 0.028 |
| Thyrotrophic Embryonic Factor | **TEF** | 1.020 | 0.028 | 1.020 | 0.804 | 1.157 | 0.211 | 1.157 | 0.056 | 0.989 | -0.016 | -1.011 | 0.895 | 1.135 | 0.182 | 1.135 | 0.094 | 0.970 | -0.044 | -1.031 | 0.708 |
| Tissue Factor Pathway Inhibitor 2 | **TFPI2** | 10.542 | 3.398 | 10.542 | <0.001 | 24.327 | 4.604 | 24.327 | <0.001 | 7.583 | 2.923 | 7.583 | <0.001 | 2.308 | 1.206 | 2.308 | <0.001 | 0.719 | -0.475 | -1.390 | <0.001 |
| Thrombomodulin | **THBD** | 1.987 | 0.990 | 1.987 | <0.001 | 8.887 | 3.152 | 8.887 | <0.001 | 1.569 | 0.650 | 1.569 | 0.003 | 4.473 | 2.161 | 4.473 | <0.001 | 0.790 | -0.340 | -1.266 | 0.061 |
| Tumor Necrosis Factor | **TNF** | 1.000 | <0.001 | 1.000 | 1.000 | 1.000 | <0.001 | 1.000 | 1.000 | 1.000 | <0.001 | 1.000 | 1.000 | 1.000 | <0.001 | 1.000 | 1.000 | 1.000 | <0.001 | 1.000 | 1.000 |
| Tumor Necrosis Factor Receptor Superfamily. Member 14 | **TNFRSF14** | 1.378 | 0.463 | 1.378 | 0.034 | 0.663 | -0.592 | -1.507 | 0.024 | 1.121 | 0.165 | 1.121 | 0.489 | 0.481 | -1.055 | -2.077 | <0.001 | 0.814 | -0.298 | -1.229 | 0.173 |
| Topoisomerase (DNA) I | **TOP1** | 0.732 | -0.451 | -1.367 | <0.001 | 0.817 | -0.292 | -1.224 | <0.001 | 0.751 | -0.413 | -1.332 | <0.001 | 1.116 | 0.159 | 1.116 | 0.055 | 1.026 | 0.037 | 1.026 | 0.672 |
| Tumor Protein P53 | **TP53** | 0.986 | -0.021 | -1.015 | 0.855 | 0.898 | -0.155 | -1.114 | 0.185 | 1.298 | 0.377 | 1.298 | 0.001 | 0.911 | -0.134 | -1.098 | 0.249 | 1.317 | 0.398 | 1.317 | <0.001 |
| Thiopurine S-Methyltransferase | **TPMT** | 0.667 | -0.584 | -1.499 | <0.001 | 0.911 | -0.135 | -1.098 | 0.245 | 0.749 | -0.417 | -1.336 | 0.001 | 1.365 | 0.449 | 1.365 | <0.001 | 1.123 | 0.167 | 1.123 | 0.228 |
| TNF Receptor-Associated Factor 2 | **TRAF2** | 1.682 | 0.750 | 1.682 | <0.001 | 1.948 | 0.962 | 1.948 | <0.001 | 0.998 | -0.003 | -1.002 | 0.988 | 1.158 | 0.211 | 1.158 | 0.174 | 0.593 | -0.754 | -1.686 | <0.001 |
| TSC22 Domain Family. Member 1 | **TSC22D1** | 1.187 | 0.248 | 1.187 | <0.001 | 0.978 | -0.032 | -1.023 | 0.412 | 1.233 | 0.302 | 1.233 | <0.001 | 0.824 | -0.280 | -1.214 | <0.001 | 1.038 | 0.054 | 1.038 | 0.140 |
| Tetratricopeptide Repeat Domain 1 | **TTC1** | 0.897 | -0.158 | -1.115 | 0.047 | 1.123 | 0.167 | 1.123 | 0.026 | 1.017 | 0.024 | 1.017 | 0.761 | 1.252 | 0.325 | 1.252 | <0.001 | 1.134 | 0.182 | 1.134 | 0.025 |
| Tubulin. Beta Class I | **TUBB** | 1.280 | 0.356 | 1.280 | <0.001 | 1.896 | 0.923 | 1.896 | <0.001 | 1.169 | 0.225 | 1.169 | <0.001 | 1.481 | 0.567 | 1.481 | <0.001 | 0.913 | -0.131 | -1.095 | <0.001 |
| Unc-51-Like Kinase 1 (C. Elegans) | **ULK1** | 1.695 | 0.762 | 1.695 | <0.001 | 1.356 | 0.439 | 1.356 | <0.001 | 1.412 | 0.497 | 1.412 | <0.001 | 0.800 | -0.322 | -1.250 | <0.001 | 0.833 | -0.264 | -1.201 | <0.001 |
| Upstream Transcription Factor 1 | **USF1** | 1.034 | 0.049 | 1.034 | 0.721 | 1.134 | 0.182 | 1.134 | 0.173 | 0.881 | -0.183 | -1.136 | 0.212 | 1.097 | 0.133 | 1.097 | 0.310 | 0.851 | -0.232 | -1.175 | 0.109 |
| Ubiquitin Specific Peptidase 5 (Isopeptidase T) | **USP5** | 1.261 | 0.334 | 1.261 | <0.001 | 1.519 | 0.604 | 1.519 | <0.001 | 0.919 | -0.122 | -1.088 | 0.036 | 1.205 | 0.269 | 1.205 | <0.001 | 0.729 | -0.456 | -1.372 | <0.001 |
| Vascular Endothelial Growth Factor A | **VEGFA** | 1.236 | 0.306 | 1.236 | <0.001 | 0.893 | -0.163 | -1.120 | <0.001 | 1.069 | 0.096 | 1.069 | 0.028 | 0.722 | -0.469 | -1.385 | <0.001 | 0.864 | -0.210 | -1.157 | <0.001 |
| Vacuolar Protein Sorting 29 Homolog (S. Cerevisiae) | **VPS29** | 0.835 | -0.261 | -1.198 | 0.035 | 0.823 | -0.282 | -1.216 | 0.023 | 0.723 | -0.467 | -1.382 | <0.001 | 0.985 | -0.021 | -1.015 | 0.870 | 0.867 | -0.207 | -1.154 | 0.134 |
| Vacuolar Protein Sorting 4 Homolog B (S. Cerevisiae) | **VPS4B** | 0.598 | -0.742 | -1.673 | <0.001 | 0.659 | -0.601 | -1.517 | <0.001 | 0.542 | -0.885 | -1.847 | <0.001 | 1.103 | 0.141 | 1.103 | 0.052 | 0.906 | -0.143 | -1.104 | 0.071 |
| WD Repeat Domain 5 | **WDR5** | 0.988 | -0.017 | -1.012 | 0.865 | 1.081 | 0.112 | 1.081 | 0.250 | 0.819 | -0.287 | -1.220 | 0.008 | 1.094 | 0.129 | 1.094 | 0.183 | 0.829 | -0.270 | -1.206 | 0.012 |
| WEE1 Homolog (S. Pombe) | **WEE1** | 0.363 | -1.463 | -2.756 | <0.001 | 0.390 | -1.357 | -2.562 | <0.001 | 0.400 | -1.322 | -2.501 | <0.001 | 1.076 | 0.106 | 1.076 | 0.282 | 1.102 | 0.140 | 1.102 | 0.165 |
| X-Box Binding Protein 1 | **XBP1** | 0.757 | -0.401 | -1.320 | <0.001 | 0.813 | -0.299 | -1.230 | <0.001 | 0.936 | -0.095 | -1.068 | 0.018 | 1.073 | 0.102 | 1.073 | 0.016 | 1.236 | 0.306 | 1.236 | <0.001 |
| Zinc Finger Protein 161 Homolog | **ZFP161** | 0.663 | -0.594 | -1.509 | 0.001 | 0.753 | -0.409 | -1.328 | 0.017 | 0.881 | -0.183 | -1.136 | 0.280 | 1.137 | 0.185 | 1.137 | 0.325 | 1.329 | 0.410 | 1.329 | 0.028 |
